# Carbon Allotrope-Based Optical Fibers for Environmental and Biological Sensing: A Review

**DOI:** 10.3390/s20072046

**Published:** 2020-04-05

**Authors:** Stephanie Hui Kit Yap, Kok Ken Chan, Swee Chuan Tjin, Ken-Tye Yong

**Affiliations:** School of Electrical and Electronic Engineering, Nanyang Technological University, 50 Nanyang Avenue, Singapore 639798, Singapore; step0031@e.ntu.edu.sg (S.H.K.Y.); kchan019@e.ntu.edu.sg (K.K.C.)

**Keywords:** graphene, carbon nanotubes, carbon dots, nanodiamonds, nanomaterials, optical fiber, sensors, nanocoating

## Abstract

Recently, carbon allotropes have received tremendous research interest and paved a new avenue for optical fiber sensing technology. Carbon allotropes exhibit unique sensing properties such as large surface to volume ratios, biocompatibility, and they can serve as molecule enrichers. Meanwhile, optical fibers possess a high degree of surface modification versatility that enables the incorporation of carbon allotropes as the functional coating for a wide range of detection tasks. Moreover, the combination of carbon allotropes and optical fibers also yields high sensitivity and specificity to monitor target molecules in the vicinity of the nanocoating surface. In this review, the development of carbon allotropes-based optical fiber sensors is studied. The first section provides an overview of four different types of carbon allotropes, including carbon nanotubes, carbon dots, graphene, and nanodiamonds. The second section discusses the synthesis approaches used to prepare these carbon allotropes, followed by some deposition techniques to functionalize the surface of the optical fiber, and the associated sensing mechanisms. Numerous applications that have benefitted from carbon allotrope-based optical fiber sensors such as temperature, strain, volatile organic compounds and biosensing applications are reviewed and summarized. Finally, a concluding section highlighting the technological deficiencies, challenges, and suggestions to overcome them is presented.

## 1. Introduction

Carbon allotropes have been extensively used in many sensing applications for targets such as temperature, pressure, magnetics, environmental pollutants, and biomolecules, either on their own or via other host-supports such as optical fibers, electrodes, and field-effect transistors [1,2,3,4]. Among these technologies, optical fiber-based sensors have attracted significant interest due to the surface versatility of silica or plastic optical fibers that allows a wide range of surface modifications. Other interesting properties, such as small dimensions and lightweight features that enable compact system design, real-time monitoring, multiplexing capabilities, and resistance to harsh environments, also offer significant advantages for the development of practical carbon allotrope-based optical fiber sensors (OFS) [5]. Carbon allotrope-based OFS exist in various system designs due to the variety of fiber structures, optical interrogation methods, and deposition techniques that can be adopted to achieve highly sensitive and selective detection of target molecules. Nevertheless, these sensors usually share a common sensing scheme, where a small portion of the guided wave energy, known as the evanescence wave, penetrates the fiber cladding and interacts with the nanocarbon coating [6]. The bindings of molecules or physical changes in the surrounding environment can be detected by the evanescence wave and contribute to a refractive index and/or optical property change that enables quantification of target molecules or physical parameters. In some instances, surface-modified carbon allotropes also permit multi-parameter detection capabilities of carbon allotrope-based OFS [7]. As such, an insight into the recent advances of carbon allotrope-based OFS can serve as a guideline to develop an effective detection approach for emerging real-world applications. Many review articles on carbon allotrope-based OFS mainly focus on a single type of carbon allotrope and the fundamental theory of carbon allotrope-based OFS [8,9]. Thus, this comprehensive review article encompasses the properties, preparation, sensing mechanisms, nanocoating deposition techniques, physical and biochemical sensing applications. This review aims to highlight the recent research work on carbon allotrope-based OFS that will serve as a reference guide for researchers to develop optimal detection approaches for physical parameters or trace level monitoring of chemical and biomolecules. Four groups of carbon allotropes, including carbon nanotubes (CNT), carbon dots (CDs), graphene, and nanodiamonds (NDs), will be studied. Commonly adopted synthesis approaches, classification of various deposition techniques for the integration of carbon allotropes as the thin film coating of OFS as well as numerous examples of these carbon allotrope-based OFS will also be outlined.

## 2. Classifications of Carbon Allotropes

### 2.1. Carbon Nanotubes

CNTs are one of the most well-known members of the nanocarbon family. CNTs can be divided into single-walled CNTs (SWCNTs) and multi-walled CNTs (MWCNTs), both of which were discovered by Iijima in 1991 and 1993, respectively [10,11]. Since then, there has been a great interest in these species due to their outstanding structural, mechanical, and electronic properties. Generally, SWCNTs comprise one layer of sp^2^-hybridized carbon atoms rolled up into a seamless cylinder with diameter and length in the nanometer and micrometer range, respectively. On the other hand, MWCNTs consist of multiple concentric CNTs with an interlayer spacing of 3.4 Å [12]. Physically, CNTs have high length-to-diameter aspect ratios, often exceeding 10,000, and therefore are one of the most anisotropic nanomaterials ever produced. Mechanically, CNTs are among the strongest and stiffest fibers, attributed to the strong sp^2^ bonds between the individual carbon atoms.

Typically, the surface properties of CNTs are the main reason for the inability of CNTs to disperse in organic or polar solvents. Even though the two ends of the CNTs exhibit oxygen-containing moieties that are generally hydrophilic, the wall that constitutes a major portion of the CNT’s surface area is hydrophobic [13,14]. Thus, CNTs are often solidly held together in bundles due to the strong van der Waals interaction. The dispersion and modification of hydrophobic CNTs are often a major challenge during the functionalization of CNTs onto the optical fibers [15]. Since CNTs in aqueous or polar solvents tend to aggregate swiftly, they are often dispersed in non-polar organic solvents such as dimethylformamide (DMF) or by modifying the CNTs with polymers or surfactants. Nevertheless, the dispersity obstacle in aqueous solution can be indirectly viewed as an advantage since optical fibers functionalized with CNTs dispersed in organic solvents enable the solvent to be easily removed by evaporation [15].

**Figure 1:**
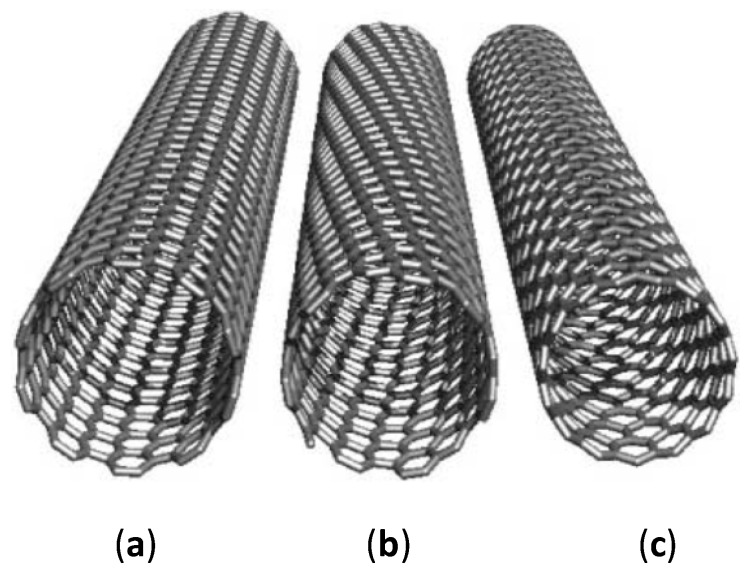
The idealized illustration of three distinctive SWCNTs with open ends. (**a**) an achiral metallic conductive armchair (10,10) SWCNT, (**b**) a chiral semi-conductive (12,7) SWCNT, (**c**) an achiral conductive zigzag (15,0) SWCNT. SWCNT in (c) is conductive as $\frac{n-m}{3}$ results in a whole number [18]. Copyright © 2002, John Wiley and sons.

The electronic properties of CNTs are highly dependent on the physical structure, such as the atomic arrangement of the carbon atoms (chirality), length, and diameter of CNTs (Figure 1). The chirality of SWCNT indicates the angle at which a graphene sheet is being rolled up, as well as the alignment of the π-orbitals. The atomic structure of CNTs can be defined in the form of a chiral vector: $\vec{c_{h}}=n\vec{a_{1}}+m\vec{a_{2}}$, where *n* and *m* can be termed as the number of steps along with the zigzag carbon bonds of the honeycomb lattice and $\vec{a_{1}}$ and $\vec{a_{2}}$ are the unit vectors. There are three different types of CNTs, namely armchair, zigzag, and chiral tubes. Armchair tubes have an equal *n* and *m* values and have a chiral angle of 30°. On the other hand, zigzag tubes have m=0, and exhibit a chiral angle of 0° while chiral tubes can exhibit any other values. The chirality is vital in determining the conductivity property of the CNTs. Conducting CNTs are in achiral and armchair configuration (*n*, *n*). Alternatively, chiral (*n*, *m*) and achiral zigzag (*n*, *0*) CNTs are semiconductors with the exception when the $\frac{n-m}{3}$ results in a whole number. As the diameter of CNTs increases, the band gap tends to reduce and can result in a zero-bandgap semiconductor [15]. Besides chirality, the properties of CNTs can also be influenced by catalytic particles and dopants [16,17], as well as functionalization of the side walls [18,19]. The physical and electronic properties have led CNTs to be integrated into sensors with various types of sensing modalities, exhibiting outstanding adaptive and sensory capabilities.

### 2.2. Carbon Dots

Fluorescent CDs were discovered accidentally during a typical gel electrophoresis purification of SWCNTs prepared via an arc-discharge procedure [20]. The fluorescent carbon nanoparticles, which later became known as CDs, are identified and separated from the carbon soot as a by-product of the arc-discharge process. CDs, also known as carbon quantum dots (CQDs) or carbon nanodots (CNDs), is another category of carbonaceous material. CDs are sometimes used interchangeably with graphene quantum dots (GQDs). However, there is some obvious physical distinctions between CDs and GQDs. GQDs refer to graphene monolayers that are fragmentized into nanosized pieces, comprising mainly of sp^2^-hybridized carbon atoms [21]. On the other hand, CDs are quasi-spherical carbon nanoparticles with a dimension below 10 nm [22,23,24]. CDs typically consist of amorphous or crystalline cores with sp^2^-hybridized carbon configuration. Some studies have also described CDs to exhibit diamond-like sp^3^ carbon configuration [25].

Surface moieties on CDs are typically introduced during the synthesis process. Different types of surface functional groups such as C=O, C–O, C–OH, and C=C can exist on the CDs surface and is highly dependent on the types of precursors used. For instance, Dong et al. [26] synthesized fluorescent CDs using citric acid and branched polyethyleneimine (BPEI) and low-temperature heating. The resultant product was found to be covered with amino-rich BPEI. These functional groups are vital for sensing applications since they can form certain coordination bonds with specific molecules and trigger some optical properties changes. The precursors used for the preparation of CDs can also introduce various types of dopant ions into CDs that can modulate the optical and sensing properties of CDs [27,28]. Shan et al. [29] employed boron-doped CDs prepared via a one-pot solvothermal synthesis using boron tribromide as the boron source and hydroquinone as the carbon precursor to sensitively detect hydrogen peroxide and glucose. Post functionalization with specific chelating groups or biomolecules is another method to endow CDs with sensitive and specific targeting capabilities [30]. In many CDs-based sensing schemes, there will be changes in fluorescence emission intensity that can be attributed to three main mechanisms, namely the inner filter effect, photo-induced electron transfer, and Forster resonance energy transfer [3].

The fluorescence property of CDs is a unique feature that is widely used in sensing applications, and the emission of CDs can be either excitation-dependent or excitation-independent [31,32,33]. For excitation-dependent CDs, the fluorescence emission wavelength can be tuned from 400 to 750 nm with a gradual increase in excitation wavelength [34]. The fluorescence intensity of the CDs can also be influenced by environmental factors such as types of solvents [35], Ph [36], temperature [37], and the concentration of CDs [38]. Interestingly, CDs also exhibit up-conversion fluorescence emission that is highly beneficial for in vivo biological applications [24,33,39,40]. Even though it is still a matter of intensive discussion, the origin of the fluorescence property is generally attributed to the quantum confinement effect, various surface functional groups, and existence of fluorophore species on the CDs surface [3].

### 2.3. Graphene

Graphene consists of two-dimensional covalently bonded monolayer carbon atoms arranged in a hexagonal network. Graphene has been long believed as a hypothetical structure until proven experimentally by the ground-breaking work of Novoselov et al. [41], which earned them the Nobel Prize in 2010. Novoselov et al. [41] developed a strategy to isolate single-layer graphene from the highly oriented pyrolytic graphite via repeated peeling using scotch tape. Since then, there has been an exponential increase in research employing graphene in optoelectronics [42], energy storage [43], energy conversion [44], and biomedical applications [45,46], due to its many unique virtues. Graphene is also an interesting candidate for sensing applications owing to its high sensitivity towards external stimuli since each carbon atom is a surface atom, thus having an extremely high surface to volume ratio [47]. Due to the delocalized π-electrons on its surface, the physical properties of graphene can also be tuned and modified to allow specific interaction with certain molecules [48].

The electrical properties of graphene also play an important role in sensing applications. Specifically, the electrical conductivity of graphene will change after the absorption of molecules on the surface of graphene because the molecules may act as electron donors or acceptors and thus, affecting the carrier concentration [49,50]. Moreover, graphene is highly conductive with low Johnson noise therefore a small variation in carrier concentration can result in a notable change in electrical conductivity. Besides, graphene has also been reported to possess surface-enhanced Raman scattering (SERS) property, enabling trace-level of target molecules to be detected by amplifying the characteristic Raman signals [51,52,53]. Serving as an alternative to noble metals such as gold or silver, graphene exhibits tunable surface plasmons at infrared and THz frequencies [54,55]. The electronic band configuration of graphene is determined by a combination of linear dispersion relation and vanishing density of states at the Fermi level in its neutral state [56,57]. As the Fermi energy deviates from the neutrality point, graphene exhibits metallic optical response, leading to the existence of plasmons. Many studies also described the advantages of graphene plasmons that include lengthy lifetime, large spatial confinement and field enhancement, as well as tunability via electrostatic grating [58].

Apart from the electronic properties, graphene presents distinctive optical properties that are widely used for sensing applications. Despite being a zero-bandgap material at pristine condition, graphene oxide (GO) that has heterogeneous functional moieties exhibits strong emission from the UV to near-infrared range [47]. The strong emission is attributed to the electronic transition between the pristine sp^2^ carbon domain and the functional moieties located at the boundaries of the GO sheets [59]. The fluorescence emission of GO can be enhanced or quenched, depending on the presence and concentration of the target molecules [60]. GO is often applied as an active material that is functionalized on an optical fiber. In many cases, the optical fiber served as the transmission medium to send the excitation signal as well as to capture the fluorescence emission to the photodetector. Therefore, an effective setup configuration that can couple maximum fluorescence emission intensity back to the fiber is critical, particularly for microstructured optical fibers [61].

### 2.4. Nanodiamonds

Diamond is long-known for its outstanding properties such as superior thermal conductivity and extreme hardness. However, nano-scaled diamonds, also known as NDs, were only discovered by Soviet scientists in the 1960s [62]. The NDs were detected in the soot after the detonation of oxygen-deficient TNT/hexogen composition in an inert environment with no additional carbon supply [63]. NDs continued to be relatively unknown until the end of the 1980s [64]. Unlike CNTs, graphene, and CDs that consist of sp^2^ graphitic carbon, NDs comprise only of sp^3^ carbon atoms and have a diamonoid-like morphology. Generally, the dimension of NDs ranges from 2 to 20 nm, which are considerably smaller than bulk diamond and diamond abrasive powders but are bigger than organic diamonoid molecules [47]. NDs tend to form aggregates, and even commercial NDs contain large NDs clusters that cannot be dispersed via ultrasonication treatment [65]. Thus, many methods have been developed to de-aggregate the NDs. Osawa et al. [66] developed two methods to break up NDs aggregate in various non-aqueous mediums. The first method is by using stirred-media milling with zirconia microbeads, capable of reducing the diameter of the NDs from 200 nm to 4–5 nm within 100 min. Despite being able to break up large aggregates, this method also wears out the beads, blades, and vessels that introduces zirconia contamination to the NDs solution. Thus, further treatment with strong acids is required to remove the zirconia nanoparticles. The second method is by using high-power ultrasonication with the assistance of zirconia beads. This bead-assisted sonic disintegration (BASD) method can reduce the size of the aggregates to a similar dimension as compared to the first method without requiring any post-treatment. Dry milling is another economical and facile approach that does not introduce any contaminant species and reduces the size of NDs aggregates from micrometer to nanometer range [67]. Pentecost employed water-soluble compounds such as sodium chloride and sucrose during the milling process that can then be removed by rinsing the NDs with water. Ultracentrifugation is another contaminant-free strategy to separate NDs into different sizes by mass and dimension, but the yield of this method to obtain single-digit NDs is very low [68].

**Figure 2:**
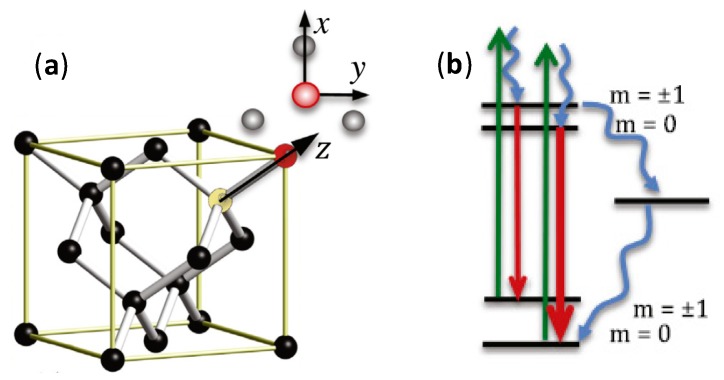
(**a**) N-V center in NDs. N and C are colored in red and black, respectively while vacancy is represented by yellow. The x and y-axes are shown on top of the z-axis. (**b**) The electronic states of NDs at room temperature. As a result of the double degeneracy of the molecular orbitals of the excited spin-triplet state, there are two orthogonal transition dipole moments. Spin-preserving PL and optical excitation are illustrated by green and red arrows, respectively [69]. Copyright © 2017, Elsevier.

The most unique property of NDs which distinguishes them from other carbon allotropes is the presence of the fluorescent defect center, known as nitrogen-vacancy (N-V) center. N-V centers have been an important characteristic for sensing applications as their PL is strong and resistant to photobleaching with an obvious zero-phonon line even at ambient temperature, electron spin triplet nature of the electronic ground state as well as the dependence of PL emission intensity on the strength of spin projection on the symmetry axis of the N-V center [69]. The N-V center is a defect in the crystal structure of NDs, as shown in Figure 2. One out of the two neighboring carbon atoms in the NDs crystalline lattice is substituted with a nitrogen atom while the other is a vacancy without any replacement atom. The two unpaired nitrogen electrons form the spin triplet ground and excited states m_s_ = 0, ±1. These states can be optically initialized, manipulated and determined at ambient temperature. After being optically excited, the N-V centers can transit between the ground and electronically excited states. The N-V centers can relax to the ground state via radiative and nonradiative pathways. Practically, the radiative transition can results in a wide PL spectrum with a zero-phonon line at 637 nm that can be employed for accurate sensing measurement [70,71]. On the other hand, the nonradiative pathway is the intersystem crossing (ISC) to the singlet states located below the excited triplet state [72]. A resonant microwave frequency can also be used as the excitation source to excite the population to m_s_ = ±1 spin level. Subsequently, non-radiative decay can take place as a result of ISC, and a decrease in PL emission can be detected. For magnetometry, the N-V center under an unknown magnetic field will produce a split between the m_s_ = ±1 spin sub-level. The electron spin resonance transition between the sublevels can be employed to determine the strength and direction of the magnetic field. In summary, Table 1 shows the properties comparison for different carbon allotropes.

## 3. Synthesis Approaches of Carbon Allotropes

Generally, carbon allotropes are prepared from carbon precursors such as graphite, organic gases, green organic compound, or volatile organic compounds (VOCs) by using various synthetic approaches to reorganize the carbon atoms. In this section, the preparation methods of CNTs, CDs, graphene, and NDs will be comprehensively discussed and reviewed.

### 3.1. Carbon Nanotubes

CNTs can be prepared via three main techniques as follow: arc-discharge method, chemical vapor deposition (CVD), and laser ablation method. The arc-discharge procedure is carried out in a vacuum chamber using two carbon electrodes as the precursor [73,74]. The chamber is filled with inert gas to expedite the carbon deposition to form MWCNTs with near-perfect morphology. A similar condition is necessary for the formation of SWCNTs but with the addition of catalysts such as Ni, Fe, Co, Pt, and Rh. On the other hand, the laser ablation technique employs an intense laser pulse to hit a carbon target in a furnace filled with inert air with the assistance of a catalyst. The bombardment of the laser beam will vaporize the carbon precursor and form a graphene film on the substrate. Despite being able to produce high-quality CNTs, both of these methods require high preparation temperatures of about 3000–4000 °C to evaporate the carbon atoms from the carbon precursor.

In contrast, the CVD technique can be used to prepare CNTs at a much lower temperature [75]. Typically, hydrocarbon gases such as methane or ethylene are channeled into a reaction chamber and will break down into reactive species at a temperature between 500–1000 °C. In the presence of metallic particles such as Ni, Fe, or Co that serve as the catalyst, the reactive species will be coated on the substrate, leading to the formation of CNTs. By adjusting the synthesis parameters and catalysts, different types of CNTs can be prepared. As the preparation requirements are lower, the CVD technique has the potential to be employed for large scale synthesis processes. Nevertheless, CNTs prepared using the CVD technique suffers from relatively high defect densities in MWCNTs, which can be due to insufficient thermal energy. Regardless of the preparation methods, the resultant CNTs are usually contaminated with carbonaceous and metallic impurities arising from the reaction process that can adversely affect the properties of CNTs. To eliminate the carbonaceous contaminants gas phase and liquid phase purification methods are introduced [76]. Gas-phase purification uses high temperature while liquid phase purification involves washing the CNTs with acidic solutions such as nitric acid or sulfuric acid. On the other hand, metallic contaminants can be removed by heating the CNTs to the evaporation temperature of the contaminant. As such, CNTs with a purity of up to 99.6% can be achieved [77].

### 3.2. Carbon Dots

In general, there are two main routes to prepare CDs, known as the top-down and bottom-up. These synthesis routes can be performed via optical, chemical, or thermal processes. In the optical synthesis method, a laser is typically used to ablate a carbon target either in water or solvents. For instance, Goncalves et al. [78] reported the preparation of CDs by irradiating carbon targets using a pulsed UV laser for 60 s. The dimension of the resultant CDs is determined by the separation between the focusing lens and the carbon target, where long distance yields CDs with larger particle sizes and vice versa. However, the resultant CDs are not fluorescent and require some post functionalization to attain fluorescent property. On the other hand, Li et al. [79] prepared CDs by carrying out the laser ablation process in different solvents such as water, ethanol, and acetone. The group discovered CDs prepared in ethanol and acetone exhibit fluorescence while no fluorescence was seen from CDs prepared in water. Therefore, the group attributed the fluorescence emission to the surface moieties generated during the synthesis process.

The chemical synthesis route generally employs strong oxidative chemicals such as concentrated sulfuric or phosphoric acid to oxidize the carbon precursors to form fluorescent CDs. In one of the reports, human hair was used as the carbon precursor and dopant source to form fluorescent CDs [27]. The human hair was added to the concentrated sulfuric acid and sonicated before being stirred at 40, 100 and 140 °C for 24 h. It was found that smaller CDs can be obtained at higher temperatures. Green precursors have also been used to produce CDs by chemical oxidation. For instance, sucrose was chemically oxidized by concentrated phosphoric acid to produce CDs [80]. The as-synthesized CDs exhibit yellow emission at 560 nm under UV excitation and is stable from pH 4 – pH 11.4. In a separate report, Hu et al. [81] prepared CDs by dehydrating and oxidizing waste frying oil with concentrated sulfuric acid. The resultant CDs exhibit uniform dimensions, partially disordered graphite-like structure, and unique pH-sensitive photoluminescence.

There are several thermal synthesis routes for the preparation of CDs, namely hydrothermal, solvothermal, direct pyrolysis, and microwave-assisted pyrolysis. Hydrothermal synthesis that employs water as the solvent is one of the simplest and cost-effective methods to prepare CDs. Solvothermal, on the other hand, uses other solvents such as ethanol and dimethylformamide [82,83]. Li et al. [83] prepared CDs using a one-pot solvothermal technique with Taixi anthracite in DMF. The resultant CDs exhibited a strong photoluminescence quantum yield of 47% and a production yield of 25.6 wt%. Direct thermal treatment and microwave-assisted heat treatment has also been widely used to produce CDs with different optical properties. Typically, the carbon precursor undergoes several processes such as dehydration, polymerization, and carbonization prior to the formation of CDs [3]. Direct thermal treatment exposes the carbon precursor to a high temperature to induce the carbonization process. However, this pyrolysis process appears less favourable since it is lengthy, and the heating duration can range up to a few hours. Thus, microwave-assisted synthesis rises as a facile alternative since it is rapid, provides uniform heating and can be executed using a domestic microwave oven [84]. For instance, Chan et al. [85] prepared nitrogen and sulfur co-doped CDs by subjecting a mixture of citric acid and thiourea in water to 6 min of microwave-assisted heat treatment. The resultant CDs were found to be responsive towards ferric ion and were employed as a sensitive and selective ferric ion sensor.

### 3.3. Graphene

Typically, the preparation of graphene by mechanical exfoliation refers to a repeated peeling process using Scotch tape to produce thin graphene flakes. In a pioneering work by Novoselov et al. [41], a highly oriented pyrolytic graphite was exposed to dry etching using oxygen plasma to produce 5 μm deep mesas. They were then placed on a photoresist and heated up to adhere to the photoresist. Subsequently, the Scotch tape was used to exfoliate layers of graphene from the graphite sheet. Thin graphene flakes of single to few layers of graphene that adhered to the photoresist were released using acetone and transferred to a silicon substrate. Despite being a simple and effective method to produce monolayer or a few layers of graphene, this technique is limited by the low production yield.

Hernandez et al. [86] introduced a liquid exfoliation technique to obtain a single to a few layers of graphene sheets by dispersion and exfoliation in solvents. Ultrasonication enables the solvent such as N-methylpyrrolidone, N,N-dimethylacetamide, γ-butyrolactone, and 1,3-dimethyl-2-imidazolidinone that have similar surface energy to graphene, to intercalate the graphite layers. Subsequent centrifugation and decantation processes produce high-quality unoxidized graphene flakes that can be used as transparent electrodes and conductive polymers. The production yield of this method is approximately 1%, which can be further increased to 7–12% by sediment recycling. The exfoliation mechanism is governed by the fact that the energy required to exfoliate graphite into single-layer graphene is countered by the solvent-graphene interaction. Nevertheless, this liquid exfoliation method suffers from incapability to control the number of graphene layers, defects, as well as difficulty to remove the residual solvents, which can have an adverse effect when used as a sensing material on OFS.

Another chemical technique to obtain graphene is by the reduction of GO. GO can be obtained by oxidizing graphite using strong oxidizing chemicals, as reported by Brodie et al. [87], Staudenmaier et al. [88], and Hummers et al. [89], among others. The oxidation process adds various types of functional groups such as carboxyl and hydroxyl moieties to the graphitic surface. GO is easily exfoliated in water and can be easily reduced and converted back into graphene. The reduction process is carried out using reducing agents such as hydrazine, hydrides, and titanium under UV illumination [90]. The main setback of this technique is that the reduction process is unable to reduce the GO completely. This process also creates defects that are unremovable via a simple annealing process and is usually of a lower quality than pure graphene. The resultant product is also sometimes referred to as reduced graphene oxide (rGO). Other methods of preparing graphene include CVD [91,92], the intercalative expansion of graphite [93], heat treatment of SiC [94,95], and epitaxial growth technique [96,97].

### 3.4. Nanodiamonds

A popular method of preparing NDs is by detonating an explosive carbon precursor such as trinitrotoluene and hexogen (1,3,5-triazinane) [98,99,100]. The detonation process takes place in an enclosed chamber supplied with inert gas or water coolant, also known as the “dry” and “wet” synthesis, respectively [101]. After the detonation process, the carbon soot contains a mixture of NDs with a diameter of 4–5 nm, other carbon allotropes, and contaminants. The weight content of NDs in the carbon soot can be as high as 75%, while the NDs yield is about 4–10% of the weight of the explosive precursor [98,99]. In a study carried out by Danilenko [102], the pressures and temperatures were found to have a significant influence on the formation of the NDs. The temperature and pressure at the Jouguet point are insufficient to produce liquid bulk carbon but are capable of producing nanosized liquid carbon. The area of liquid carbon is moved to a lower temperature for nanocarbon while the area of NDs stability is marginally shifted to a higher pressure. As such, it is implied that the NDs are formed by homogeneous nucleation in supersaturated carbon vapor by condensation and crystallization of liquid carbon. A major drawback of this method is that the NDs tends to aggregate and are not dispersible in organic solvents or water. To make matters worse, the ND aggregates are often covered in a layer of graphitic material and further complicates the dispersion of NDs.

The NDs aggregates can be broken down by mechanical milling or ultrasonication. Krüger et al. [103] managed to reduce the size of NDs aggregate with diameters of 100–200 nm, 2–3 μm, and 20–30 μm to NDs with a dimension of 4–5 nm by stirred-media milling with microscale ceramic beads. The group speculated that the milling process is mainly based on the shearing action within the fast-turbulent flow.

Meanwhile, Ozawa et al. [104] introduced a bead-assisted high-power ultrasonication technique to break up NDs aggregates. The resultant nanosized NDs can be dispersed in various types of polar solvents such as water, dimethyl sulfoxide (DMSO), and ethanol. Nevertheless, these methods also introduce contaminants originating from the beads, blades, and vessels. Purification procedures such as reflux treatment in acid and centrifugation are required to remove the impurities.

The pulsed laser has also been reported to produce nanoscale NDs. Amans et al. [105] used a pulsed laser to ablate a graphite target in water. The resultant NDs have a dimension of 5–15 nm but is covered by a graphitic-like structure with a thickness between 3–4 nm. Findings from a separate study indicate the dimension of the NDs can be controlled by manipulating the laser light source [106]. With the same power density, a short pulse width laser will obtain single-crystal NDs with size between 3–4 nm, while long pulse width laser will obtain particles larger than 4 nm. In another report, Kumar et al. [107] developed a microplasma process to prepare NDs at near ambient environment. The NDs were homogeneously nucleated by dissociating ethanol vapor and quickly quenched with a reaction duration of less than 1 ms to achieve particle dimension in the nanoscale range. With the assistance of hydrogen gas, the non-diamond phase is removed while the diamond phase is retained and stabilized, resulting in a high-purity NDs. The resultant NDs have an average size of 3 nm, which is in accordance with theoretical calculations. Table 2 summarizes the synthesis approaches for each type of carbon allotrope.

## 4. Preparation Techniques of Carbon Allotrope-Based Optical Fiber Sensors

For chemical or biosensing applications, the coating conditions (i.e., temperature, pH, and duration), thickness, and uniformity are among the important factors in determining the sensor performance. For example, a non-uniform and thick coating is usually undesirable since it may lead to poor sensing performance. Furthermore, the response and recovery time of carbon allotrope-based OFS are equally affected by the coating thickness due to the adsorption dynamics between the target molecules and the nanocoating. To achieve a stable, repeatable, and high sensitivity carbon allotrope-based OFS, it is important to optimize the coating parameters by adopting suitable deposition techniques. In the following section, several established techniques for the deposition of carbon allotropes onto OFS will be discussed.

### 4.1. Langmuir-Blodgett (LB)

**Figure 3:**
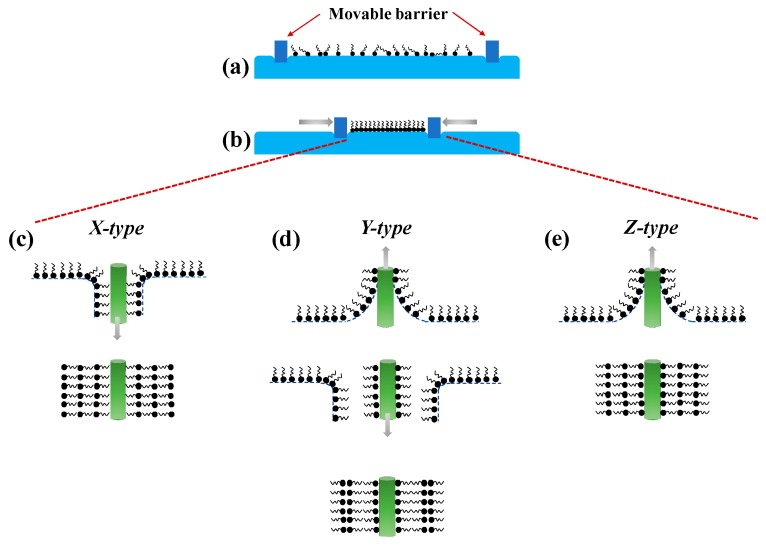
Langmuir-Blodgett film deposition scheme. (**a**) Spreading of molecules to the surface of sub-phase, (**b**) surface compression with constant pressure to yield a condensed and stable monolayer film, (**c**) *X-type* LB film deposition, (**d**) *Y-type* LB film deposition, and (**e**) *Z-type* LB film deposition.

Irving Langmuir and Katharine Blodgett first introduced the Langmuir-Blodgett (LB) technique for the deposition of nanocoating onto a solid substrate. The deposition process is commonly performed at room temperature and usually involves amphiphilic molecules with hydrophobic tails and hydrophilic heads. Using this method, the optical fiber is usually prepared to have a hydrophilic surface and is placed in the sub-phase. Next, the receptors with water-insoluble amphiphilic molecules are prepared in volatile organic solvents and applied to the surface of the sub-phase. The molecules are oriented such that the hydrophilic part stays in the water while the hydrophobic part is facing upwards (Figure 3a), creating a floating monolayer of molecules in an arranged manner on the surface of sub-phase. Next, controlled compression is applied to the surface to form a condensed and stable monolayer film (Figure 3b) for the subsequent deposition onto the optical fiber that is vertically raised through the sub-phase. If multiple layers of coating are desired, the substrate is returned into the sub-phase to create head-to-head and tail-to-tail stack layer pattern, commonly known as *Y-type* (Figure 3d). Conversely, to obtain a monolayer coating, the deposition techniques known as *X-type* (monolayer transferred during downstroke only) (Figure 3c) or *Z-type* (monolayer transferred during upstroke only) (Figure 3e) can be carried out [108].

The LB surface coating technique offers precise control over the deposition thickness, approximately 1–3 nm for each layer on the planar substrate. Deposition onto a single-mode optical fiber, for instance, is able to achieve 2.6 nm for each deposited molecular layer [109]. In order to achieve the desired film thickness, the amount of surface tension being applied and the material concentration are some of the important parameters that need to be considered [110]. Compare to other deposition techniques, fabrication of LB film on the optical fiber can be as simple as requiring only one chemical compound, given it an added value in terms of homogeneity. On the flip side, this could also mean that only a limited number of chemical compounds can be used with this technique. The fabricated LB film also suffers from poor thermal stability [111]. Moreover, this deposition process is tedious, slow, and requires skilled executor and sophisticated instrument to control the surface tension. For these reasons, the LB technique appears to be less popular unless nanometric precision of coating thickness is desirable.

### 4.2. Layer-by-Layer Electrostatic Self-Assembly (LbL-ESA)

LbL-ESA technique was first demonstrated by Decher and co-workers in preparing a multi-layered film on a solid substrate by alternate exposure to anionic and cationic polyelectrolyte with immediate adsorption of the oppositely charged ions [112]. This approach has attracted tremendous interest particularly in the field of surface material engineering due to its outstanding merits such as uniformity, stability, simplicity, and excellent controllability of the coating thickness at the nanometer scale. The driving factor to this deposition technique premises upon the electrostatic interaction between two materials of opposite charges. Typically, multi-layered nanocoating using LbL-ESA is usually performed at room temperature, and is independent of the size and shape of the substrate unlike the LB approach [113]. The fabrication of LbL-ESA begins with treating the substrate to obtain a negatively charged surface. The treatment varies according to the types of substrates, for instance, common silica-based substrates such as glass slide and silica optical fiber are usually done via piranha solution, a mixture of concentrated sulfuric acid (H_2_SO_4_) and 30% hydrogen peroxide (H_2_O_2_) at 3:1 v/v ratio. The strong dehydrating power of the H_2_SO_4_ and oxidizing power of H_2_O_2_ remove the organic residues from the surface, followed by generating a dense layer of hydroxyl groups (–OH), making it highly hydrophilic and favorable for subsequent electrostatic interaction with polycation electrolyte. Similarly, the immersion of plastic optical fiber with poly(methyl methacrylate) (PMMA) core material into 1M H_2_SO_4_ helps to develop carboxylic groups (–COOH) by reducing the methyl ester groups of PMMA [114]. After obtaining a negatively charged surface, the optical fiber is then immersed into a polycation solution for a sufficient amount of time to allow for molecules to adsorb. Next, the optical fiber will be washed thoroughly with deionized water and dried before immersing it into a polyanion electrolyte-containing solution. This will yield one bilayer LbL-ESA film, and the process can be repeated for several cycles to achieve the desired multi-layered thin-film structure.

In general, LbL-ESA process takes place in an aqueous solution but slowly diversify to nonpolar solvents due to the discovery of novel nanomaterials. Although the deposition mechanism remains unchanged, Lindgren et al. [115] recently reported a new insight into the significant role of the solvent towards the effectiveness of electrostatic assembly. The study explains different types of solvents may alter the electrostatic force between the interacting particles and surface, from attractive to repulsive or vice versa. The type of interaction is dependent on the permittivity of the particles and solvent. Briefly, a solvent with a large dielectric constant that is more polarizable than both interacting particles and surface promotes repulsive interaction, while a solvent with a small dielectric constant promotes attractive electrostatic force [116]. Therefore, it is rational for one to include the polarization effect of the solvent in designing the deposition system. Due to its vast applicability to most of the optical fiber platforms and the broad availability of molecules, LbL-ESA can be employed for entire surface area or end-face deposition on the optical fiber sensor probe to suit different sensing schemes. However, low molecular weight molecules alone are incapable of being assembled directly onto the optical fiber using the LbL-ESA method due to deficiency of charged groups in these molecules and are likely to face a substantial amount of loss in the rinsing step. To counter this problem, deposition with a single or combination of polyelectrolytes with long alkyl chain such as PAA, poly(allylamine hydrochloride) (PAH), polyethylenimine (PEI), etc. onto the substrate is preferred. For instance, Goncalves et al. [117] developed an Hg^2+^ sensor using a tapered tip silica fiber by immobilizing PEI as the polycation electrolyte followed by depositing CDs at the end tip of the sensor probe. Similarly, Alberto et al. [118] reported a GO-coated tilted Bragg grating prepared using the LbL-ESA method. Alberto et al. [118] first treated the optical fiber with sodium hydroxide to produce a negatively charged surface for subsequent deposition of poly(diallyldimethyammonium chloride) (PDDA) and poly(sodium 4-styrene-sulfonate) in an alternate manner. PDDA that served as the polycation electrolyte was coated to the external layer of the optical fiber before the deposition of the negatively charged GO. Besides polyelectrolyte-carbon allotropes multi-layered film, the LbL-ESA technique is also adopted for the development of metal oxide-polyelectrolyte-carbon allotrope films on optical fiber substrates. In a work done by Hernaez et al. [119], the deposition of PEI/GO multi-layered films onto a tin oxide-coated multimode fiber has significantly enhanced its sensitivity for ethanol sensing by 20% and 210% for one and four bilayers of PEI/GO, respectively. Henceforth, LbL-ESA method of fabricating carbon-allotrope coatings have become one of the most favorable deposition technique to develop a wide range of OFS.

### 4.3. Chemical Vapor Deposition (CVD)

The fundamental of plasma-assisted vapor deposition lies in the activation of a precursor in a glow discharge (i.e., plasma) environment. The growth of a thin-film using this method generally exhibits less contamination as compared to other wet techniques [120]. In a generic case, carbon nanomaterial thin film produced using the CVD process is formed from the chemical reaction of gaseous reactants in the close vicinity of a lightly heated substrate (~20–50 °C). Briefly, the initiator and the target material, usually in liquid form, are vaporized by either heating or reducing the air pressure, followed by passing it to a vacuum chamber where the substrate is placed. The initiator functions to accelerate the film growth rate and finally, the target material will be deposited on the cold substrate. The thickness and the refractive index of the CVD film can be easily adjusted by altering the pressure and temperature of the deposition process. In many generic cases, synthesis of carbon allotropes such as graphene thin film using the CVD approach usually involves reaction gases like methane and dilute hydrogen environment, on a copper foil as a catalyst substrate at over 1000 °C [6]. Transfer of the thin film can be done in several ways. To attach the graphene thin film onto the fiber structure, graphene is transferred to a low refractive index substrate, such as MgF_2_, followed by adhering onto the optical fiber via van der Waals bond [121]. If wrapping the graphene thin film surrounding the fiber structure is desired, one way to achieve this is by spin-coating a layer of PMMA on the surface of the graphene forming a PMMA/graphene/copper hybrid. Next, the copper under graphene is removed using iron (III) chloride solvent followed by wrapping the PMMA/carbon nanomaterial thin film on the fiber. Finally, the PMMA is removed with acetone leaving only the graphene thin film on the fiber.

Nevertheless, the merits of CVD are obvious, particular its ability to fabricate dense and amorphous films, and more importantly, good uniformity [122]. Despite these advantages, CVD is not with no limitations. Temperature or UV sensitive materials are not suitable for these techniques, thus there is a limited number of materials that can be deposited on the optical fiber using this approach. Moreover, high accuracy and high-resolution instruments are required to regulate important parameters such as temperature, pressure, current, and others that may significantly affect the reproducibility of the nanocoating. For these reasons, the CVD setup is costly and may be inaccessible to some laboratories due to its high operating cost.

### 4.4. Optical Deposition

The optical deposition method utilizes the light guided by an optical fiber to draw the sensing material in close proximity to the optical fiber surface, and finally, depositing onto the external surface of the optical fiber. Commonly, the optical deposition technique involves dispersing the sensing material into solvents such as ethanol, isopropanol, dimethylformamide, and others, followed by immersing the fiber into the mixture. Kashiwagi et al. [123] reported the coating of CNTs onto the end facet and tapered region of the optical microfiber via the optical deposition method. Together with the optical forces from the injection of light into the sensing material-dispersed solution, the Brownian motion of the sensing material became highly oriented, and the swirl and convection tend to draw them toward the surface of optical fiber. Kashiwagi et al. [123] also deduced that the trapping of this sensing material on the microfiber surface is jointly resulted from the optical tweezer effect due to optical intensity diversion in the solution caused by the evanescent field of the optical microfiber. Since the size of the CNTs is much smaller than the wavelength of the light, the CNTs are treated as a point dipole. Two forces acting on this dipole, the scattering force that pushes the particle along the light propagation and the Lorentz force which moves the particle toward the region of higher optical intensity [124,125]. Centered on this principle, the evanescent field of the optical microfiber that had the optical intensity diversion may trap the CNTs by optical tweezer effect and consequently, immobilized them onto the surface of the desired area [126].

Generally, the film thickness produced using optical deposition technique is controllable by adjusting the injected light power and the deposition time. Besides, in-situ monitoring of the transmitted or reflected power is often performed to monitor the insertion loss caused by the deposited sensing material. Ideally, it is recommended that the insertion loss falls within 3 to 5 dB. Overall, the simple and economical process of the optical deposition technique is capable of achieving the area-selective deposition of sensing material.

### 4.5. Crosslinking

In the context of conjugation, a crosslinker is used to mediate the attachment of one molecule to another, usually through the covalent bond to create a complex comprising of both molecules linked together. Generally, the design of the conjugation process is dependent on the reactive groups present on the reactive crosslinking agents and the functional groups present on the target molecules. The conjugation process is unfeasible without the availability and chemical compatibility of both reactive and functional groups. Examples of these functional groups include amine, thiol, carboxylate, aldehyde, and hydroxyl. Meanwhile, some reactive groups that are often employed in the conjugation process include isothiocyanate, isocyanates, NHS ester, maleimide, and glutaraldehyde [127].

**Figure 4:**
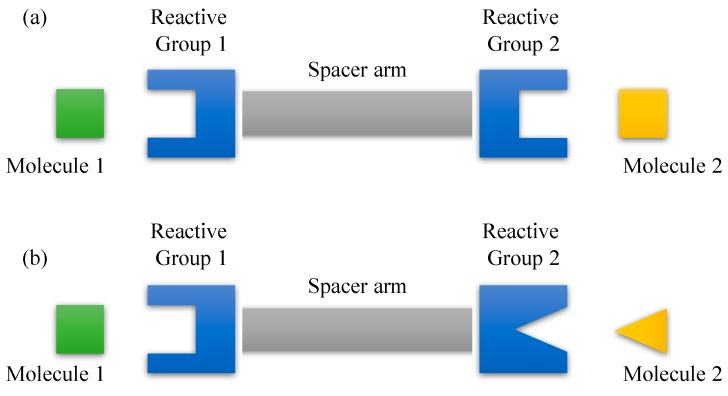
General design of crosslinking agent. (**a**) Homobifunctional crosslinker with identical reactive groups at the end of spacer arm and (**b**) heterofunctional crosslinker with two different reactive groups at the either end of spacer arm.

In many instances, the final conjugate complex is bound by a crosslinker that introduces foreign chemical components to the molecules being crosslinked. The first crosslinking agent introduced for the conjugation of macromolecules, known as the homobifunctional crosslinker consists of bireactive compounds of the same functionality at both ends of the spacer arm (Figure 4a) [128]. The use of a homobifunctional crosslinker in a one-step conjugation protocol, however, provides the least control over the final product of a conjugation reaction and may yield a broad range of poorly defined conjugates [129]. This is because when crosslinking two molecules, for instance, the homobifunctional crosslinker first reacts with either one of the molecules, forming an active intermediate. Ideally, this activated molecule may crosslink with the second molecule, however, it may also react intramolecularly with other functional groups on part of its own. To circumvent this, a two-step conjugation protocol may alleviate the problem. This can be done by removing the excess crosslinker and byproducts before introducing the second molecule to the activated molecule to allow the final conjugation reaction to take place. This solution, however, may raise another problem where the activated molecule experience degradation before the second phase of crosslinking commence due to hydrolysis phenomena. Moreover, chances of the problem associated with the one-step conjugation protocol may persist in the two-step conjugation protocol since the first molecule may crosslink with itself long before the introduction of the second molecule. Thereafter, another type of crosslinker known as heterobifunctional crosslinking agent that contains two different reactive groups at the end of the spacer arm (Figure 4b) is introduced for targeted coupling between two different functional targets on macromolecules. Heterobifunctional crosslinker exhibits the ability to yield direct crosslinking reaction to selected parts of target molecules and thus, warrant better control over the resultant product of the conjugation reaction.

Another commonly used crosslinker would be the zero-length crosslinker, also known as the smallest crosslinking agent available to perform a conjugation process. A zero-length crosslinker allows one atom of a molecule to attach covalently to another atom of a second molecule with no additional intervening atoms or spacer in between the bond. This is advantageous, since in some cases, the presence of these intervening linkers or spacers in between the established bond may cause cross-reactivity with undesired reactive/functional groups. Carbodiimide such as 1-ethyl-3-(3-dimethylaminopropyl) carbodiimide hydrochloride (EDC), is most popularly adopted for conjugating substances containing carboxylate to molecules containing amine functional groups. While EDC alone can be used in a one-step conjugation protocol, the efficiency of the conjugation process can be enhanced through the use of N-hydroxysuccinimide (NHS) that increases the solubility and stability of the intermediate before conjugating with the targeted amine groups [130,131].

For silica-based optical fiber, the foundation layer to facilitate the surface functionalization process is solely dependent on the hydroxyl groups present on the external surface. However, these hydroxyl groups are rather weak and insufficient for conjugation with other functional groups via crosslinking. Therefore, many works reported an additional step of performing oxygen plasma or acid treatment such as using a piranha solution to form a dense and active layer of hydroxyl groups for subsequent crosslinking use. Some also reported the use of silane coupling agents to create amine or carboxylate terminal end groups to ease the conjugation reaction with carbon allotropes via zero-length crosslinker [132].

### 4.6. Drop-Casting

Drop casting is another simple yet economical approach of depositing sensing material onto the surface of the optical fiber. In a typical manner, the as-prepared carbon allotrope is dispersed in a volatile solvent. Meanwhile, the bare optical fiber is cleaned with an alcohol-based solvent followed by annealing in an oven before initiating the deposition process. Next, the carbon allotrope solution will be drop-casted at the desired deposition area on the fiber and left undisturbed at the ambient environment for the solvent to evaporate naturally. This process can be repeated depending on the desired amount of deposited material. Alternatively, different concentrations of carbon allotrope solution can be used to achieve the desired coating thickness. Post deposition process usually followed by annealing the optical fiber for a second time to enhance the coating adhesion. Carbon allotrope-based OFS fabricated using this deposition technique is vastly reported in the past few years [133,134,135].

## 5. Sensing Mechanisms of Carbon Allotrope-Based Optical Fiber Sensors

Many research groups have utilized the unique properties of carbon allotropes to develop OFS for diverse applications. This section will mainly discuss the different sensing mechanisms employed in carbon allotrope-based OFS.

### 5.1. Thermo-Optic

CNTs, NDs, and graphene are known to exhibit excellent thermal conductivity attributable to their C-C covalent bond and phonon scattering characteristics [8]. The high thermal conductivity of these nanomaterials makes them interesting candidates and can be exploited as a temperature-sensitive material for temperature sensing. For instance, Zhang et al. [1] developed an all-fiber temperature sensor based on rGO. The group described that as the temperature increases, the availability of thermally excited electrons-holes also increases. This changes the Fermi-Dirac distribution of electrons in the rGOs that sequentially decreases the dynamic conductivity. Theoretically, the real part of dynamic conductivity affects the amount of light absorbed by the intraband and interband transitions in the rGO. Therefore, a rise in temperature will reduce the amount of light being absorbed and hence, resulting in a reduction of transmission loss and an increase in transmitted optical power. As a result, temperature measurement can be attained using rGO film on an optical fiber. Despite the availability of different carbon allotrope-based platforms such as resistive-based sensors that exploit the thermal conductivity for sensory applications, there are still some major setbacks that restrict its translation for practical application. For instance, resistive-based sensors are costly, energy-intensive and often require elevated temperature to introduce metal oxide layers as part of the device configuration [136]. Contrarily, the configuration of temperature sensors using optical fiber is relatively simple, and the carbon allotrope-based sensing layers can be introduced via many facile strategies as described in Section 4. However, careful selection of light wavelength and source power are needed to minimize the potential of self-heating cause by the absorption of the injected light source that can affect the measurement accuracy.

### 5.2. Surface Plasmon Resonance

In a typical surface plasmon resonance (SPR)-based OFS, precious metals such as gold or silver that serve as surface plasmon materials are coated on the optical fiber to form SPR structure. Gold coating is beneficial as it introduces a larger resonance shift to the changes in refractive index at the sensing layer while silver coating which exhibits a smaller SPR curve width will result in a higher signal to noise ratio. However, the procedure to introduce these metallic coatings on an optical fiber is complicated and costly. For example, silver is easily oxidized when exposed to oxygen in ambient air, elevated temperature, or water vapor due to their poor chemical stability and resulted in the oxidation of silver to silver oxide [8,137]. This will affect the sensor’s reproducibility and thus remains impractical for real-life sensing. To overcome this, a bilayer metallic coating configuration that comprises of a silver and gold (outer layer) coating may solve the problem for sensing applications [138]. However, careful optimization of the layer thickness is vital to achieve high signal to noise ratio as well as optimal sensitivity of the proposed sensor. Thus, carbon allotropes, especially graphene, rises as a potential alternative due to their superior properties and resistance towards oxidation. Furthermore, it has also been reported that graphene can enhance the SPR signal [139]. By depositing a layer of graphene on the optical fiber, a fiber-graphene interface that supports charge density oscillation upon light excitation can be obtained. It should be noted that the excitation light should have an identical polarization state as well as matching momentum and wave vector to the surface plasmon [140]. As a result, a resonance is generated at a particular wavelength, and a sharp wavelength dip, also identified as resonance wavelength can be measured in the output spectrum, and the presence of target molecules can be detected as a shift in the resonance wavelength. On top of this, the concentration of the target molecules can also be detected and quantified by correlating to the amount of shift in the resonance wavelength.

### 5.3. Fluorescence

CDs, NDs, and graphene are among the fluorescence carbon allotropes that are commonly integrated into an optical fiber-based fluorescence sensor. The emission of these nanomaterial ranges from the UV and visible spectrum under various excitation wavelengths. In general, a fluorescence sensor operates based on the perturbation in optical characteristics such as the fluorescence intensity in the presence of the target molecules. This process can take place based on several mechanisms, such as photoinduced electron transfer (PET) or fluorescence resonance electron transfer (FRET) [3]. PET can be described when a new complex is formed between an electron donor and an electron acceptor. Upon excitation, the new complex will return to the ground state without the emission of photons. On the other hand, FRET is an energy transfer process that occurs between a donor molecule and an acceptor molecule via dipole-dipole interactions [141]. The energy received will excite the donor molecule to the lowest unoccupied molecular orbital (LUMO). Subsequently, the energy will be transferred to the acceptor molecule while the donor molecule relaxes to the ground state. For this to happen, the absorption spectrum of the acceptor molecule requires an overlap with the emission spectrum of the donor molecule. Furthermore, since the energy transfer efficiency is inversely proportional to the sixth power of the distance between the donor and acceptor molecules, both donor and acceptor molecules need to be in close proximity to allow the occurrence of this process.

### 5.4. Molecular Adsorption

Carbon allotropes such as graphene possess a high density of hexagonal ring structure that can be functionalized on the optical fiber surface to absorb molecules such as gas molecules, heavy metal ions, and organic pollutants. The number of molecules being absorbed can be correlated to the changes in the refractive index and detected using an optical fiber. For example, evanescence wave-based optical fiber is well known for its sensitivity to the perturbation in the surrounding refractive index. In a typical manner, light launched into the core of the fiber first propagates as fundamental mode, HE_11_. As the light reaches the sensing region, a substantial amount of light energy will be coupled into the next high-order mode, HE_12_, as a result of the morphology and local refractive index change. Unlike HE_11_ mode, the HE_12_ mode is not confined within the core but exposed to the outer surface and become cladding guided. As the light travels down the fiber, a second coupling occurs between the HE_11_ and the HE_12_ modes and generates a phase difference between the two modes that will result in a modal interference spectrum governed by the ${I=I}_{1}+I_{2}+2\sqrt{I_{1}I_{2}}\cos\phi$ [142,143]. In the event where target molecules are absorbed on the carbon allotropes, a perturbation of localized refractive index on the sensing region will occur and can be detected as a spectrum wavelength shift in the output signal of the fiber. An example can be seen from a work reported by An et al. [144] using a D-shaped fiber coated with a thin gold film followed by a layer of graphene to measure the refractive index range from 1.38 to 1.39. The presented sensor showed a maximum sensitivity of 4391 nm/RIU at a resolution of 2.28 × 10^−5^ using the wavelength interrogation method. Likewise, Fu et al. also simulated a D-shaped fiber for refractive index sensing in the range of 1.33 to 1.39 [145]. The D-shaped fiber is designed such that silver nano-columns coated with graphene layers are deposited on the side polished area to enhance the sensitivity to the surrounding refractive index change. Maximum refractive index response sensitivity can reach up to 8860.93 nm/RIU when the diameter of the silver nano-column is fixed 90 nm and 23 layers of graphene, each layer with a thickness of 0.34, is coated on each silver nano-column. In another study, two different etched fiber Bragg grating (FBG) sensors coated with SWCNTs and GO, respectively, demonstrated high specificity to protein concanavalin A (Con A) via the mannose-functionalized poly(propyl ether imine) dendrimers attached to the coated sensors [146]. Even in the presence of interfering proteins such as bovine serum albumin and lectin peanut agglutinin, SWCNTs and GO coated etched FBG sensors showed great selectivity to Con A and are able to achieve LODs of 1 nM and 500 pM, and affinity constant of ~4 × 10^7^ M^−1^ and ~3 × 10^8^ M^−1^, respectively. Overall, these studies have widely proven the possibility of using carbon allotrope-based OFS not just in chemical or environmental sensing but also in biological sensing applications.

## 6. Sensing Applications of Carbon Allotrope-Based Optical Fiber Sensors

### 6.1. Humidity

Humidity sensing is vastly employed for domestic and industrial applications. For instance, humidity sensors are often employed in smart buildings, food processing plants, and microelectronics industries. Relative humidity (RH) is defined as the amount of water vapor present in the air, expressing the ratio of the actual moisture in the air to the maximum amount of moisture that the air can retain at that temperature and is quantified in terms of percentage. Optical fiber-based humidity sensor rises as an alternative solution to overcome the drawbacks of conventional humidity sensors such as hygrometer and psychrometer that exhibit long response time and suffer from electromagnetic interference. Moreover, the versatility of optical fibers to different functional nanocoatings has led to the integration with carbon allotropes for the development of optical fiber-based humidity sensors. Shivananju et al. [147] reported an etched fiber Bragg grating (FBG) coated with CNTs at the etched region for humidity sensing. Due to the interaction between water molecules and the CNTs coating, the effective refractive index surrounding the core will change, resulting in a shift of the Bragg wavelength and a sensitivity of 31 pm/%RH within a linear detection range from 20–90 %RH. Mohamed et al. [148] prepared an optical microfiber coated with MWCNTs slurry using the drop-casting technique. The fabricated sensor demonstrated a linear detection range from 45 to 80 %RH and an improvement of 1.3 times (5.17 μW/%RH) when compared to a bare tapered fiber. Alternatively, doping of MWCNTs onto a PMMA microfiber to form a thin layer of nanocoating on the optical microfiber for RH sensing was reported by Isa et al. [149]. The MWCNTs/PMMA functional coating increases the index contrast between the microfiber core and the surrounding air cladding causing more water molecules to be adsorbed on the sensing surface. Consequently, more electrons are transferred from the water molecules to MWCNTs, diminishing the available holes in MWCNTs and thus, decreasing the output optical power of the MWCNTs/PMMA microfiber sensor. The sensor showed a good linear detection range between 45 to 80 %RH and sensitivity of 0.3341 dBm/%RH, demonstrating an approximately 4-fold sensitivity improvement over a undoped PMMA microfiber sensor. Similarly, Ma et al. [150] also developed a CNT/polyvinyl alcohol (PVA) coated at the end tip of the thin core fiber (TCF) for RH detection. The as-developed sensor demonstrated good reversibility and output stability after 12 consecutive exposures to different RH levels. Moreover, the sensor showed a good linear detection range from 70 to 86 %RH with a measured sensitivity of 0.4573 dB/%RH.

On the other hand, GO-based OFS have also attracted significant interest for their prominent use in RH sensing. Gao et al. [151] presented a hollow-core fiber coated with rGO in which the sensing mechanism is based upon the adsorption of water molecules on the rGO surface that serves as electron acceptors. Along with the adsorption of water molecules, the surface charge carrier density of rGO will increase, further inducing a change in the chemical potential and dynamic conductivity of the rGO. The changes in these parameters will then influence the effective refractive index of the rGO and hence, altering the output signal of the sensor. The sensitivity of the fabricated rGO-based hollow-core fiber sensor was found to increase with increasing sensor length, and a maximum sensitivity of 0.229 dB/%RH was achieved within the linear detection range from 60–90 %RH at the fiber length of 12.6 cm. Furthermore, the sensor showed good reversibility and was unaffected by the surrounding temperature fluctuation. When tested with human breath alone, a swift response time of 5.2 s and a recovery time of 8.1 s was recorded. Xing et al. [152] prepared rGO nanosheet-coated polystyrene (PS) microsphere via a thermodynamically-driven hetero-coagulation approach to create a three-dimensional graphene network (3-DGN) coating surrounding the taper waist region of an optical microfiber. It is interesting to note that PS microsphere alone is hydrophobic in nature. However, upon functionalized with rGO, the chemically active defect sites of rGO that exhibit hydrophilic functional groups such as carboxylic and carbonyl groups are likely to absorb water molecules present in the surrounding environment. Moreover, the constructed 3-DGN was able to achieve much higher sensitivity as compared to single rGO or GO nanocoating. The measured results obtained from the rGO/PS-coated optical microfiber exhibited a sensitivity of −0.224 dB/%RH and −4.118 dB/%RH for detection range 50.5–70.6 %RH and 79.5–85 %RH, correspondingly. Overall, Table 3 summarizes all carbon allotrope-based RH OFS and their respective sensing performance [134,147,148,149,150,151,152,153,154,155,156,157,158,159,160,161,162].

### 6.2. Temperature and Pressure

An rGO-based side polished fiber sensor using the refractive index change scheme for temperature sensing was developed by Zhang et al. [1]. Briefly, when the surrounding temperature increase, the concentration of thermally excited electrons-holes increases and the change in the Fermi-Dirac distribution of electrons in the rGO will reduce the real part of its dynamic conductivity. Since the real part of the dynamic conductivity correlates to the light absorption induced by intra and interband transitions in the rGO, reduction in the real part of the dynamic conductivity will consequently decrease the light absorption and reduce the transmission loss of the rGO-coated side polished fiber [163]. In other words, as the surrounding temperature increases, the transmitted optical power of the rGO-coated side polished fiber also increases. Therefore, a linear relationship of surrounding temperature as a function of output transmitted optical power was obtained for the rGO-coated side polished fiber within the range of −7.8 to 77 °C with a maximum sensitivity of 0.134 dB °C^−1^ was achieved. Other optical fiber structures such as etched FBG [164] and suspended core hollow fiber [165] coated with rGO have also been employed to demonstrate temperature sensing capability. On the other hand, Sun et al. [166] reported a graphene-coated optical microfiber temperature sensor constructed with a thin graphene film adhered onto the optical microfiber surface via the strong evanescent field and the electrostatic force. To reduce the insertion loss, the graphene thin film was transferred to MgF_2_ with a lower refractive index before introducing them to the optical microfiber surface. As the surrounding temperature fluctuates, the graphene thin film and MgF_2_ substrate alter the effective refractive index of the optical microfiber. Since the thermo-optic coefficient of MgF_2_ (3.2 × 10^−7^ °C^−1^) is much smaller than graphene (7.385 × 10^−6^ °C^−1^), the effect of the temperature change on MgF_2_ substrate is negligible. Good linearity and temperature sensitivity of 0.1018 dB °C^−1^ and 0.1052 dB °C^−1^ were measured when surrounding temperature increase and decrease, respectively, in steps of 5 °C between 30–80 °C. In a similar context, Wang et al. [167] proposed the inclusion of polydimethylsiloxane (PDMS) to produce a PDMS-graphene pliable composite film wrapping around the microfiber ring resonator. Similar to the MgF_2_ substrate, PDMS exhibits low refractive index and high thermal stability, making it a good candidate to work with graphene to achieve better performance of the temperature sensor. Moreover, PDMS is a highly transparent film with a high degree of flexibility. As a result, these features enable very close contact between the graphene and the fiber that helps to improve the temperature response of the proposed sensor. The proposed sensor exhibits excellent temperature sensitivity of 0.541 dB °C^−1^ under an incremental temperature environment and 0.542 dB °C^−1^ under gradually decreasing temperature environment for the temperature range of 30–60 °C.

**Figure 5:**
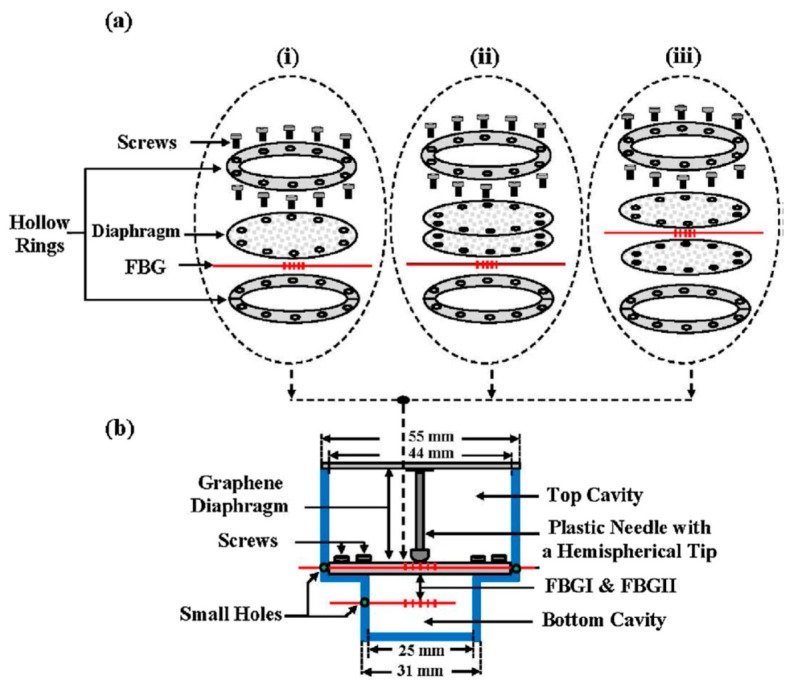
Experimental architecture: (**a**) diaphragm incorporated FBG positioned between two hollow rings, (i) a single sheet of graphene diaphragm on the top of FBG, (ii) two sheets of graphene on the top of FBG, (iii) two sheets of graphene between FBG called sandwich layer. (**b**) Sandwich layers of the sensor head structure [168]. Copyright © 2016, Elsevier B.V.

Some studies also reported the simple fabrication of graphene diaphragm integrated into an optical fiber sensor for both temperature and pressure sensing. For instance, Ameen et al. [168] proposed FBG-based temperature and water level sensors of different configurations, as shown in Figure 5. The study revealed that FBGI is responsive to water levels that are associated with the hydrostatic pressure, while FBGII is sensitive to the surrounding temperature. For both parameter measurements, increasing the number of graphene diaphragm layers, each with a thickness of 25 µm, tends to decrease the sensing performance owing to the reduction in graphene diaphragm elasticity. This attributes to the fact that for a thinner diaphragm, stronger diaphragm deflection is expected in response to the external pressure, while thicker diaphragm will bend less under the same amount of applied pressure leading to a smaller responsivity. Thus, a single-layer graphene diaphragm was found to deliver the best detection sensitivity of 13.31 pm °C^−1^ for temperature sensing within the range of 27 to 75 °C and 253.21 pm kPa^−1^ for pressure or water level sensing. However, this single-layer graphene diaphragm is only able to resist up to a maximum of 9.81 kPa that is equivalent to 100 cm of water level. To enhance the detection range sensor configuration as illustrated in Figure 5a(iii) has a higher tolerance up to 135 cm of water level without damaging the diaphragm but with a lower sensitivity of 99.18 pm kPa^−1^. Alternatively, Dong et al. [7] presented a simpler design of a Fabry-Perot interferometer with an integrated FBG to measure pressure and temperature changes simultaneously. Graphene sheet was coated onto the end facet of the fiber ferrule via van der Waals reaction to form a reflecting surface of a sealed Fabry-Perot microcavity. The study revealed that as the surrounding temperature increases, the cavity length increases together with the red shifting of the resonant wavelength. However, for pressure increase, only the cavity length decreases while the resonant wavelength remains unchanged. Therefore, by using matrix inversion calculation, the variation in surrounding pressure and temperature can be identified simultaneously. The proposed sensor was reported to exhibit temperature and pressure sensitivity of 306.2 nm °C^−1^ and 501.4 nm kPa^−1^, respectively.

Cui et al. [169] recently reported an analytical model that can predict the critical diaphragm thickness wherein the responsivity of the pressure sensor is independent on the elasticity property when the diaphragm thickness is below the critical thickness value. In other words, further reduction of the diaphragm thickness will not help to improve the sensor’s sensitivity if diaphragm thickness is much smaller than the diaphragm deflection. Redesigning the cavity shape other than cylindrical appears to be a possible solution for this, where the sensitivity of the sensor can be enhanced if the cavity shape design yields a larger cavity volume without altering the resonator length and diaphragm radius. The denouement of this study has raised the importance of placing more effort on optimizing cavity shape rather than opting for smaller single-layer diaphragm thickness that will complicate the sensor design. Table 4 and Table 5 summarize some of the research works related to temperature and pressure detection using carbon allotrope-based OFS.

### 6.3. Other Physical Parameter Sensing Applications

**Figure 6:**
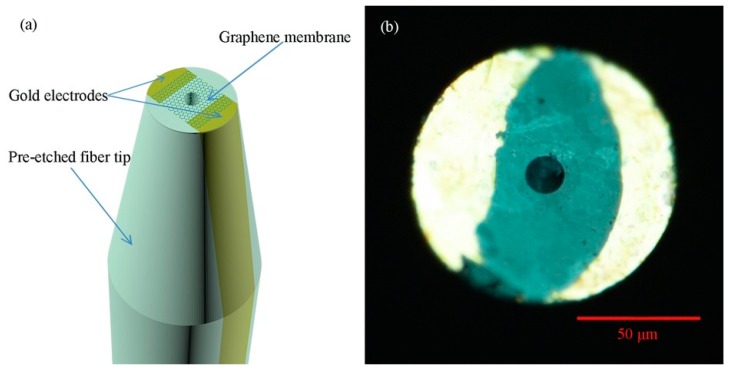
Schematic of (**a**) Pre-etched fiber tip coated with graphene membrane that covers the hole and two gold electrodes and (**b**) optical image of the tip’s end facet [177]. Copyright © 2015, WILEY-VCH Verlag GmbH & Co. KGaA.

Besides the above-mentioned physical parameters, current, acoustic, wind speed, and magnetic are some other physical parameters that can adopt carbon allotrope-based OFS to execute the measurement. For example, Zheng et al. [177] prepared a graphene membrane coated fiber tip probe sensor for current sensing, as shown in Figure 6. Having a gold electrode and graphene membrane covering the end face of the etched fiber, electric current was coupled to the fiber sensor via two contact pads. The functional coating was heated up due to the applied current, and thus, the linear temperature change of the graphene membrane can be correlated to the square of current applied. Due to the negative thermal expansion coefficient of the graphene membrane, the graphene membrane will contract uniformly with increasing temperature, and further increasing the cavity length. These physical changes were reflected as a resonance wavelength shift in the reflectance output spectrum. The proposed sensor exhibited a current sensitivity of 2.2 × 10^5^ nm/A^2^ within the detection range from 0 to 2 mA and a short response time of 0.25 s. Even though higher sensitivity can be achieved by reducing the size of the graphene membrane to expedite the heating process, the operating range of the developed sensor remains limited to 2 mA to avoid damage to the fiber probe sensor.

The practicability of graphene to be used as a deflectable diaphragm to sense pressure change has also inspired its feasible use for acoustic sensing. Ma et al. [176] designed a Fabry-Perot interferometer for acoustic sensing using a 125 μm multilayer graphene diaphragm with a thickness of 100 nm coated at the end face of the single-mode fiber. The sensor was placed in front of a speaker that acted as the acoustic pressure source, and the reflectance output spectrum was measured. The sensor demonstrated approximately 1100 nm/kPa of acoustic pressure sensitivity and noise-limited detectable pressure of approximately 60 μPA/Hz^1/2^ at 10 kHz. The study further revealed that the sensitivity of the fiber sensor is dependent on the alignment angle of which the speaker was placed. Maximum sensitivity was achieved when the fiber sensor is aligned to the central axis of the speaker. Meanwhile, the sensitivity was found to decrease as the speaker is moved to a certain angle from the central point of the fiber sensor. Tan et al. also investigated the effect of graphene diaphragm’s diameter by comparing the acoustic sensing performance of two Fabry-Perot interferometers each formed by immobilizing a 100 nm thick graphene diaphragm of 2.5 mm and 125 μm diameter respectively onto the end face of a single-mode fiber [178]. The work revealed that graphene diaphragm with a larger diameter exhibited 31 times improvement in sensitivity that agrees well with the linear deflection model.

Hot wire fiber anemometer is another promising sector demonstrating the potential use of OFS for measurement of wind speed in the coal mine, power transmission, and agricultural industries. In general, OFS based on hot wire anemometer correlates the measured cooling rate or temperature variation to the wind flow rate. Typically, the fiber sensor that incorporates nanomaterials with good heat conversion coefficients such as metal films and CNTs is usually heated up and subjected to wind flow. The amount of temperature drop measured by the sensor can then be used to approximate the wind flow rate. Based on the aforementioned detection scheme, Zhang et al. [179] proposed a simple hot wire fiber anemometer using SWCNTs coated tilted FBG. A pump laser of center wavelength 1550 nm acted as the heating and broadband light source to obtain a transmission spectrum of the tilted FBG were combined using a 3 dB coupler and launched into the core of the probe sensor. The coupled pumped light interacted with and absorbed by the SWCNTs film, which then raised the local temperature of the fiber sensor. When the heated sensor is subjected to a wind field, the cooling rate associated with the wind speed is detected by a wavelength shift in the resonance peak of the tilted FBG. Further, it was discovered that higher pump power of laser source, larger tilted angle of probe sensor, and thicker SWCNTs coating can enhance the wind speed sensitivity. However, considering the chirped effect of grating-inscribed fiber that may weaken the sensor response and harder dissipation of heat generated within the thick SWCNTs, pump power of 97.76 mW and SWCNTs coating of 1.6 μm thick was found to be optimal. The developed sensor was found to achieve a wind speed sensitivity of −0.3667 nm/(m/s) at the wind speed of 1 m/s.

Liu et al. [180] also worked on fabricating Au/SWCNT-based titled FBG anemometer sensor with an LOD of 0.05 m/s on account to the SPR effect of the gold coating that provides sensitivity enhancement. A similar study has also been performed by Liu et al. [181] who reported a SWCNT-based long period grating anemometer sensor with a simpler sensing setup and a sensitivity of 102.5 pm/(m/s) at the wind speed of 1 m/s.

**Figure 7:**
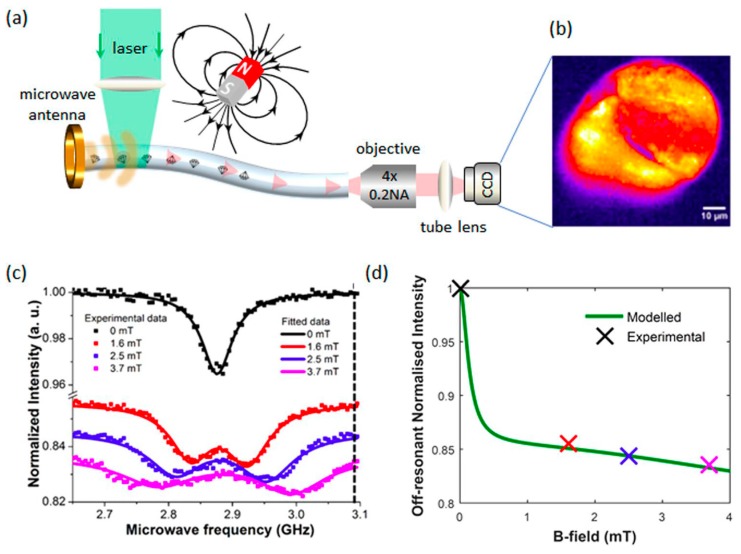
Magnetically sensitive ND-doped tellurite glass fiber. (**a**) Schematic diagram of the optically detected magnetic resonance using ND-doped tellurite glass fiber. (**b**) Image of the NV emission from the fiber end face captured using an sCMOS camera. (**c**) Output spectra of the sensor with varying magnetic field strength. (**d**) Theoretical and experimental results for the off-resonant fluorescence intensity versus magnetic field [182]. Copyright © 2018, Springer Nature.

Although OFS is well-known for its immunity to electromagnetic interference, Ruan et al. [182] highlighted the advantages of magnetically-sensitive OFS in their work that provide a cheaper, robust, and ambient alternative for remote magnetic sensing use. This work reported a novel technique to fabricate an ND-doped tellurite glass fiber for magnetic field sensing to provide an isolated sensing platform since the magnetic sensitive materials are immobilized in the tellurite glass matrix, as shown in Figure 7a. The NDs used in this work were developed via a non-detonation approach with an average particle size of 40–50 nm and a substantial density of negatively-charged NV defects were introduced to the NDs by means of high energy irradiation with 2 MeV electrons followed by annealing under vacuum at 800 °C to mobilize the vacancies to the intrinsic nitrogen. The negatively charged NV centers in the ND exhibits unique electron-spin properties in which they possess non-zero electronic spin and favorable energy level structure that support spin polarization to occur due to manipulation with magnetic field that can be interrogated by visible light. Since a single NV center enables magnetic surface mapping with nanometric spatial resolution, this resulted in NDs with a high density of NV centers to offer extremely high magnetometric sensitivity [183]. The ability to sense the magnetic field using the ND-doped tellurite glass fiber is highly dependent on the fluorescence emission of the NV centers that can be observed due to the control of the ground state population [184]. Figure 7b illustrates the inhomogeneous distribution of optical image captured from the fiber end face that validated the emission was originated from the collection of spatially distributed incoherent sources of the NVs in the doped ND. When an external magnetic field was applied, a decrease in the fluorescence emission intensity at 637 nm was observed (Figure 7c). This is attributed to the mixing of ‘bright’ (m_s_ = 0) and ‘dark’ (m_s_ = 1) spin states that occur when spins and external magnetic field are misaligned [185]. Apart from the emission wavelength, the overall reduction in the fluorescence intensity with the increasing magnetic field was also observed at off-resonant fluorescence intensity, as seen in Figure 7d. The presented sensor demonstrated an inherently magnetic field sensitivity of 10 μT/Hz^1/2^. Bai et al. [186] recently published a novel ND-doped lead silicate glass fiber that not only yielded an improved magnetic field sensitivity of 350 nT/Hz^1/2^, but also introduce a new avenue of coupling fluorescence emission from ND to guided modes of conventional step-index optical fiber.

### 6.4. Heavy Metal Ions

Heavy metal pollution in the environment arises from both natural phenomena and anthropogenic activities such as volcanic eruptions, weathering, soil erosion, mining, coal burning, and other industrial, and agricultural production activities. Although some of the heavy metals like iron, copper, and zinc are essential nutrients for various biochemical and physiological functions, exposures to a high level of these metal ions can result in lethal effects such as organ failure and slowing the progression of physical and neurological degenerative processes [4]. Furthermore, heavy metals are persistent, bioaccumulate, and have negative impacts on the ecological health that may lead to contamination of food chains. For these reasons, heavy metal sensing is critical, particularly for water and soil quality monitoring applications. To date, many research efforts are channeled to the development of highly sensitive and selective sensors, ranging from portable handheld sensors to paper-based heavy metal sensors [187,188,189]. OFS incorporating carbon allotropes as the sensing element to detect heavy metal ions is particularly interesting since it does not only retain the advantages of conventional OFS but also improves the detection sensitivity, owing to the large surface to volume ratio of these carbon allotropes. Alwahib et al. [190] proposed a nickel ion sensor using a gold-coated D-shaped optical fiber that incorporates a functional coating comprising of rGO/Fe_2_O_3_ composite. The rGO composite is rich in hydroxyl, OH^−^, and carboxyl, COOH^−^ groups, thus, creating a strong affinity to the positive charge Pb^2+^ ions due to the electrostatic interaction between them. It was further discovered that the sensor operates on a dual-mode interrogation scheme, intensity change, and resonant wavelength shift, depending on the target detection range. At lower Pb^2+^ concentration range, output intensity was found to be more responsive as compared to output resonant wavelength shift that was constrained by the low resolution. Thus, an LOD of 0.3 μg/L and 1 mg/L were obtained for a linear detection range of 0–1 mg/L and 1–15 mg/L, correspondingly. Yao et al. [191] also developed a “FRET on fiber” sensing platform to detect various analytes, including Cd^2+^. The sensor was prepared by immobilizing partially rGO (prGO) onto the fiber, followed by Rhodamine 6G (R6G). In the absence of Cd^2+^, no fluorescence scattering is observed from the R6G due to the quenching effect of prGO. However, when Cd^2+^ ions are present, R6G will be released due to the higher affinity of prGO to Cd^2+^, hence the fluorescence intensity will be restored. A pulsed laser of center wavelength 532 nm is focused on the sensing region, and the output fluorescence signals are captured by optical lenses and measured using a spectrometer. In addition to the fluorescence signal detection, binding of Cd^2+^ onto the prGO also induces refractive index change surrounding the prGO sensing region that is reflected as an output wavelength shift at wavelengths between 1510–1590 nm. This suggests that the developed sensor can be used as a fluorometric or an interferometric sensor. Moreover, the established sensing platform exhibits an ultra-high resolution of wavelength detection of 1 pm, achieving an LOD of 1.2 nM for Cd^2+^ detection.

Apart from graphene, a pioneering work conducted by Goncalves et al. [117] focused on fabricating CDs via laser ablation method followed by functionalization with NH_2_-polyethylene glycol (PEG_200_) and N-acetyl-L-cysteine. The end product was then deposited onto the fiber end tip using the LbL coating technique for Hg^2+^ detection in water. It was deduced that the responsivity of the sensor towards the presence of Hg^2+^ is primarily contributed by the special coordination chemistry between the thiol groups belong to the N-acetyl-L-cysteine and Hg^2+^. This occurrence is detected when the fluorescence emission of CDs measured by the optical fiber decrease gradually due to the fluorescence quenching by Hg^2+^. To achieve optimal sensing performance, the proposed sensor was prepared in different configurations. It was discovered that the sensor with 6 layers of CDs/PEG_200_/N-acetyl-L-cysteine performed the best with an LOD of 0.01 µM and a linear detection range of 0.01 to 2.69 µM. Moreover, the fabricated sensor also showed high physical and chemical stability where no signal degradation or residual fluorescence was observed despite multiple cycles of drying and hydration. In another interesting work, Yap et al. [132] developed an evanescent wave-based optical microfiber sensor that uses CDs as the chelator for detecting Fe^3+^ in aqueous and biological samples. The as-synthesized nitrogen- and sulfur-co-doped CDs were prepared via a microwave-assisted pyrolysis approach and is the first instance which reports the use of CDs as a chelator to perform metal ion sensing at the solid-liquid interface without relying on its fluorescence properties. The abundance of hydroxyl, amine, and carboxyl groups present on the surface of the CDs yield specific coordination to Fe^3+^ that promotes an excellent detection limit of 0.77 µg/L and good selectivity to Fe^3+^ despite the presence of other interfering ions in aqueous samples. Quantification of Fe^3+^ concentration was obtained by correlating the amount of output wavelength shift that arises from the effective refractive index change of the sensing region due to the chelation of Fe^3+^ by the CDs. The authors also demonstrated the feasibility of integrating the CD-functionalized optical microfiber sensor with a portable optical interrogation system to facilitate on-site application use. When tested against Fe^3+^ spiked samples such as tap water, biological buffers, and animal serums, the test results were in good agreement with ICPMS results, further confirming the reliability and practicability of the proposed sensor for Fe^3+^ detection. Other studies that involved the integration of carbon-allotropes with OFS can be found at [78,192,193].

### 6.5. Alcohol

Alcohol such as ethanol is colorless, flammable, and is commonly employed in extensive industrial applications ranging from household products, biomedical supplies, beverage and chemical industries. Ethanol sensors that are simple, practical, and reproducible have become important for these manufacturing industries to ensure consistent production and a safe working environment. Even though exposures to ethanol will not cause any deleterious effects, it will still result in minor health impacts such as headache, drowsiness, eye irritation, and difficulty in breathing. In view of this, Girei et al. [194] presented a tapered multimode fiber coated with GO for ethanol detection in water. The graphene was prepared using the electrochemical exfoliation of graphite rod using sodium dodecyl benzene sulfonate, followed by a simplified Hummers method to synthesized GO. The end product was then drop-casted onto the taper waist of the fiber and annealed to strengthen the coating. To quantitate the measured ethanol concentration, the absorbance response within the visible wavelength range of the sensor was found to increase linearly with increasing ethanol concentration from 5 to 40%. It is believed that the ethanol detection occurs on account of the electron transfer mechanism between the graphene layer and oxygen molecules that consequently induces a change in the coating surface index. Girei et al. [194] also prepared two different fiber sensors, one coated with graphene while another with GO for comparison purposes. Tapered fiber sensor coated with GO demonstrated an approximately two-fold increase in sensitivity with a sensitivity of 1.33 a.u./% as compared to graphene-coated tapered OFS. Nevertheless, both sensors showed good reproducibility after three consecutive cycles of exposure to 5% of ethanol solvent, indicating the high reversibility and stability of the coating. The response and recovery time for graphene-coated fibers were 15 and 18 s, respectively. Meanwhile, for GO-coated fiber, the recorded time was 25 and 12 s correspondingly.

**Figure 8:**
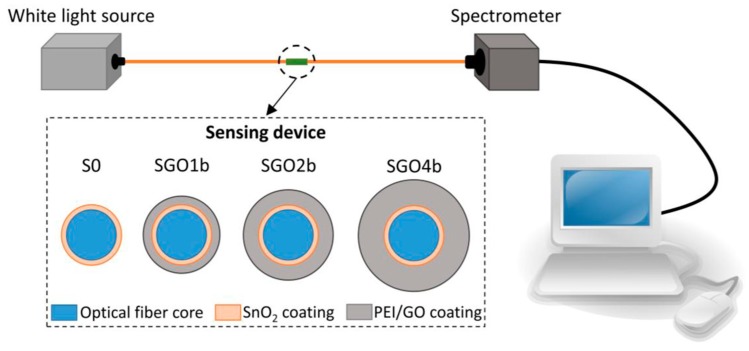
Ethanol sensing experimental setup using SnO_2_/PEI/GO coated fiber [119]. Copyright © 2018, MDPI.

On the other hand, Aziz et al. [195] developed an Ag/rGO coated tapered multimode fiber to perform ethanol sensing via the LSPR operation principle. Similar to an earlier report by Girei et al. [194], when the fiber sensor was introduced to increasing ethanol concentration from 1 to 100%, the output absorption spectrum in the range of 350 to 800 nm showed an increase in intensity. The authors also prepared an rGO-only coated tapered multimode fiber and conducted the same test for ethanol sensing. It was later found that the Ag/rGO coated fiber exhibited higher sensitivity than rGO-only coated fiber. This is attributed to the presence of optical and catalytically active Ag nanoparticles that are sensitive to the local refractive index change induced by the binding of ethanol molecules, and thus, enhances the output signal. Aside from attaining an LOD of 1%, the Ag/rGO coated fiber sensor also recorded a response time of 11 s and a rapid recovery time of only 6 s. Hernaez et al. [119] also studied lossy mode resonance (LMR)-based SnO_2_/PEI/GO coated multimode fiber for ethanol sensing (Figure 8). The LMRs yield an absorption band/resonance peak at a specific wavelength in the transmission spectrum and is modulated by the variation in the surrounding refractive index stimulated by the ethanol molecules being absorbed onto the surface coating.

For the sake of comparison, fiber sensors of one, two, and four bilayers of PEI/GO were prepared to study the effect of coating thickness towards the sensing performance. With reference to a bare SnO_2_ fiber, the study revealed that a significant sensitivity enhancement was observed in the sensor with 4 bilayers coating as the amount of resonant wavelength shift of the LMR peak was the highest (176.47%) among other sensors for ethanol concentration ranging from 0 to 100%. The amount of sensitivity enhancement is even higher (210%) when the range of ethanol concentration of interest is reduced to only 0–40%. Furthermore, the dynamic response of the sensor when exposed to 4 consecutive cycles of 40% ethanol showed the sensor exhibits good reusability and had a swift response and recovery time of 1 s and 2.75 s, respectively.

CNTs have also been reported for aqueous ethanol sensing when Shabaneh et al. [196] fabricated a tapered multimode fiber tip coated with CNTs using the drop cast approach. The CNTs coating was uniformly deposited on the end facet of the fiber probe sensor, and the thickness was approximately 370 nm. The reflectance signal of the fiber sensor was analyzed and found to decline with increasing concentration of ethanol solution. The fact that the CNTs are sensitive to ethanol can be explained since the pristine CNT used in this work was treated with nitric acid that serves as an oxidizing agent to introduce covalent attachment of carboxylic groups, −COOH, on the CNT surface. In the presence of ethanol molecules, hydrogen bonds are formed between the −COOH groups of the CNTs and the −OH groups belong to the ethanol molecules. As a result, this dipole-dipole interaction will cause a change in the surface index of the CNT coating and was reflected as a decrease in the reflectance spectrum. The developed sensors demonstrated good reproducibility and achieved ethanol sensitivity of 0.1441 a.u./%. The response and recovery time of the sensor was measured as 50 and 53 s, respectively. In summary, Table 6 collates information on all carbon nanomaterial-based optical fiber ethanol sensors and their respective sensing performance [197].

### 6.6. Ammonia and Volatile Organic Compounds

Ammonia is widely employed in the production of explosives, fertilizers, and as an industrial refrigerant even though it is known to be a highly toxic colorless gas, and inhalation of only a small amount of ammonia vapor may create potential hazards to human health. On the other hand, volatile organic compounds (VOCs) are categorized as carbon compounds with a low boiling point, high vapor pressure, toxic, and may result in serious environmental problems such as the formation of photochemical ozone smog, greenhouse effects, stratospheric ozone depletion, and others. The contribution of VOCs rising from anthropogenic emission has been increasing significantly due to the development of the industry. Common sources of VOCs emissions are often linked to the exploitation, transport, and usage of fossil fuels. Furthermore, non-point source VOCs caused by leakage, evaporation during production, and storage activities are equally significant and harder to control. In recent years, great efforts have been made to establish efficient solutions to mitigate ammonia and VOCs pollution as well as reliable sensors to monitor the level of ammonia and VOCs concentration in the air. The crossover between the attractive properties of carbon allotropes and the benefits offered by optical fibers has led to an attractive platform for ammonia and VOCs sensing. This can be seen from a study conducted by Kavinkumar and Manivannan, who synthesized Ag- decorated GO sheets by reducing the AgNO_3_ with vitamin C in the presence of GO [202]. The abundance of epoxide and hydroxyl functional groups present on the GO enabled the in-situ formation of positively charged Ag nanoparticles to be adsorbed onto the negatively charged GO surface via the strong electrostatic interaction. Subsequently, when ammonia molecules (NH_3_) interact with the Ag-decorated GO sheets, electron transfer reaction occurs between the two entities, making Ag-decorated GO sheets naturally responsive to the NH_3_ vapor. Premised on this sensing mechanism, the as-developed Ag-decorated GO sheets-coated multimode fiber sensor was able to achieve a sensitivity of 0.17 counts/ppm for NH_3_ concentration ranging from 0 to 500 ppm.

The same year, Kavinkumar and Manivannan also prepared a GO-MWCNTs-coated multimode fiber sensor for VOCs detection at room temperature [203]. The unique combination of GO and MWCNTs provides an improved gas sensing performance due to the large surface to volume ratio and high amount of oxygen functional groups of GO composites that served as the binding sites for the target gas molecules. The variation of output intensity of the fiber sensor is largely affected by the physical adsorption of gas molecules on the surface of the GO-MWCNTs coated on the outer surface of the fiber sensor. The adsorbed gas molecules act as an electron donor to the functional coating and subsequently lead to an increase in charge density of the functional coating that concomitantly changes the refractive index of the modified fiber. The proposed sensor is capable of detecting NH_3_, ethanol, methanol vapor with sensitivities of 0.41, 0.36, and 0.23 counts/ppm, respectively.

Embedding CNTs within host-matrices of foreign material is a way to improve the gas sensing performance such as enhanced sensitivity and selectivity and extension of detection capabilities to variables of gas molecules. Consales et al. [204] designed and coated a cadmium arachidate(CdA)/SWCNTs composite onto the distal end of a single-mode fiber using the LB deposition technique and capable of detecting xylene, toluene, ethanol, and isopropanol. Even though the incorporation of CdA successfully yields detection sensitivity enhancement over the SWCNTs-coated fiber, the CdA matrix also affects the adsorption dynamics of the gas molecules onto the functional coating and this lengthens the response time of the sensor. Recently, Zhang et al. [205] prepared graphene-doped tin oxide nanocomposites for methane sensing using a side polished single-mode fiber as the sensing platform. Typically, SnO_2_ is an n-type semiconductor material that contains electrons as its majority carrier. When methane molecules are in contact with SnO_2_, the adsorbed molecules will diffuse freely across the thin film and loses its motion energy. Electrons from the methane molecules are transferred into SnO_2_, turning into positive ions that lead to the increase of charge carriers in SnO_2_ and consequently increase the conductivity and effective refractive index of the thin film. Nevertheless, the resistivity of pristine SnO_2_ thin film alone is very high such that the amount of carrier concentration is hard to control since it is determined by the number of oxygen vacancies. For this reason, doping of graphene into the SnO_2_ thin film appears to be a way to increase high carrier concentration for SnO_2_ as well as improving the conductivity of the thin film that gives rise to an enhanced methane molecules sensitivity. The transmittance intensity of the output signal was found to increase with increasing methane concentration (0–55%) to which the fiber was exposed to, and a sensitivity of 200 a.u./% was obtained. Table 7 summarizes all recent advances in the fabrication of carbon nanomaterial-based OFS for VOC detection.

### 6.7. Other Biomolecule Sensing

By exploiting the evanescent wave of an optical fiber, carbon allotropes can serve as a functional coating capable of detecting bio-analytes and alter the output light properties for interrogation purposes. For instance, Qiu et al. [220] synthesized graphene film using the CVD technique and transferred directly onto the tapered PMMA core section of the plastic optical fiber. The graphene layer serves as a good molecule enricher or an absorbable film supported by the plastic fiber that can firmly absorb glucose molecules onto its surface and alters the output light intensity. A gradual decrease of output light intensity was observed when tested against 1–40% of glucose concentration. Similarly, Jiang et al. [221] also demonstrated a U-bent plastic optical fiber sensor for glucose sensing. Briefly, PVA/graphene/AgNPs thin film was coated onto the U-bent plastic optical fiber via the dip-coating method. The study investigated the effect of bent inner diameter and found that the refractive index sensitivity of the fiber sensor decreases with an increasing inner diameter, thus, U-bent plastic optical fiber with 5 mm inner bent diameter was chosen for subsequent glucose detection study. The sensitivity of the as-developed LSPR-based fiber sensor against glucose solutions of concentration ranging from 1.25 to 20% resulted in glucose sensitivity of approximately 0.9375 nm/%. Sharma and Gupta presented a graphene-based chalcogenide fiber optic sensor for hemoglobin detection in human blood samples [222]. The evanescent wave of the optical fiber interacts with the graphene monolayer that serves as a bio-enricher material in the proposed sensor. In the presence of hemoglobin of various concentrations, a significant portion of light was absorbed and output light intensity was reduced. At 1000 nm wavelength, an LOD of 18 µg/dL and hemoglobin sensitivity of 6.71 × 10^−4^ per g/dL were obtained. On top of that, the detection of uric acid [223], dopamine [224], human IgG [2] have been reported using graphene and CNTs-based OFS using the evanescent wave detecting scheme.

## 7. Future Outlook

The studies on carbon allotrope-based OFS have provided us with a deeper insight into the current trend of carbon allotrope-based sensing schemes and applications. Undoubtedly, carbon is an element with the widest and most diverse types of structure. Many carbon allotropes with different morphologies can be formed by the various arrangement of sp^2^ and sp^3^ carbon atoms, resulting in many different nanostructures with unique properties. Even though carbon allotropes have immense commercial potential in the sensing industries, there are still some challenges that need to be addressed before the commercialization of carbon allotrope-based sensors is possible. Firstly, extensive investigation into the preparation of the carbon allotropes is required to achieve uniform and reproducible nanostructures. For example, CDs can be prepared via various carbon precursors and can be doped with single or multiple dopant atoms to modulate the optical properties [225]. However, intensive studies are required to warrant a deeper understanding of the formation mechanism as well as the effects of the precursor types and synthesis methods on the resultant CDs to gain better control of the optimization work for sensing performance enhancement. Next, the stability of carbon allotropes is also a matter of concern. rGO, for instance, can form via chemically or thermally reduced route from GO. For this reason, the operating temperature of the GO-based OFS is often limited to prevent the undesired reduction process of the GO structure. Thus, even though it is advantageous for OFS to operate at high temperatures to allow quicker molecules adsorption and desorption process, most of the reported GO-based OFS were tested at ambient temperature. Besides, the sensitivity and selectivity of carbon allotrope-based sensors can also be further enhanced by proper surface treatment or ion doping. In a work by Ham et al. [226], it was reported that oxygen plasma treatment on CNTs improved the immuno-sensing detection limit by almost 1000 times when compared to the standard ELISA assay. On the other hand, non-treated CNTs exhibit no detectable signal. These results show that the oxygen-containing moieties introduced during the oxygen plasma process have a favorable effect on the sensing performance of the CNT-based sensors.

The integration of these carbon allotropes with optical fiber has given rise to many different OFS that are capable of detecting a wide range of molecules/parameters such as toxic gas, biomolecules, heavy metal ions, humidity, pressure, and others. As compared to other currently available technologies for sensing industries, carbon allotrope-based OFS sensing platform is advantageous in the aspect of resistance to corrosion, immune to electromagnetic interference, distributed and multiplexing sensing capabilities, and large dynamic operating range. However, the development of the OFS sensing platform is slightly more sophisticated than other sensing technologies such as electrochemical-based sensors that are excellent in terms of generating an output signal that can be used directly for hardware circuit, or microwave-based sensors such as microwave resonators that can also be easily linked to wireless sensing network platform due to the compatible operating frequencies. Table 8 summarizes the advantages and limitations of optical fiber sensing technology as compared to other conventional methods such as electrochemical, acoustic, microwave, and mass spectroscopy.

In summary, although OFS have humongous potential for sensing applications, there are still some obvious limitations that need to be addressed. Firstly, cross-sensitivity to factors such as temperature and pressure can significantly affect the sensing performance of the OFS. Thus, careful design of the sensory system is required to minimize interferences. One may implement an array of OFS, each functionalized with different sensing materials that exhibit different affinity levels to a group of target molecules in order to obtain multivariable responses which allow statistical method such as linear discriminant analysis to be done to improve sensor’s selectivity [227]. A multiplexing system also enables the translation of the existing optical fiber sensing platform for distributed sensing applications. This can be done by integrating commercial optical frequency domain reflectometry system to spatially discriminate the measurand at different locations along a fiber length.

Secondly, traditional optical fiber measurement requires an optical spectrometer that is costly and bulky because it comprises many optical parts such as mirrors, beam splitters, and other assemblies to deliver broad wavelength coverage. Not only that these characters make the entire sensing system difficult for on-site measurement, but they may also appear to be redundant for some applications. In fact, a narrower wavelength span requirement is likely to ease the realization of a compact optical interrogator for the development of a portable OFS. Certainly, intensity interrogation will be much easier to implement given the fact that a simple and cost-effective photodetector is sufficient to deliver the output signal in electrical form. If wavelength interrogation is unavoidable, several novel interrogator designs involving the use of post-inscription of fiber gratings in the optical sensor probe can be used [228,229], but at the cost of complicated fabrication procedures and the possibility of affecting the sensor performance.

Alternatively, one may incorporate fiber Bragg grating fibers into the design of the optical sensing system, serving as line markers that measure the light intensity at the pre-determined wavelengths of the output spectrum to estimate the amount of wavelength shift [187].

In short, future research works requires the synergistic effort from the material science groups and optical fiber technologists to integrate the benefits of carbon allotropes with optical fiber as well as to incorporate multiplexing capabilities, distributed sensing and Internet of Thing (IoT) to produce the next generation of carbon allotrope-based OFS.

## Figures and Tables

**Table 1:** Comparison of carbon allotropes properties.

Type of Carbon Allotrope	Properties
Carbon Nanotubes	High length to diameter aspect ratios Requires functionalization to reduce the hydrophobicity Tends to aggregate due to strong van der Waals interaction Electronic properties are dependent on the chirality, length and diameter of CNTs.
Carbon Dots	Have tunable fluorescence Can be excitation dependent or excitation independent Have numerous surface moieties on the surface Doping and functionalization can improve sensing capabilities Flexibility in selecting the starting precursors
Graphene	High surface to volume ratio Conductivity of graphene can be influenced by the attachment of analyte Can be employed for various sensing modalities based on their electronic, SERS and fluorescence characteristics
Nanodiamonds	Fluorescence based on the nitrogen-vacancy center Resistant to photobleaching Fluorescence is sensitive to magnetic field changes

**Table 2:** Comparison of synthesis approaches for carbon allotropes.

Types of Carbon Allotropes	Synthesis Strategy	Advantages	Disadvantages
Carbon nanotubes	Arc-discharge	Can provide high-quality CNTs Simple apparatus High reproducibility	High synthesis temperature Contaminated with impurities
	Chemical vapor deposition	Lower synthesis temperature Uses hydrocarbon gas as the precursors Different types of CNTs can be prepared by varying the precursors.	Contaminated with impurities Requires many optimization processes from the catalyst preparation to the CVD reaction
	Laser ablation	Higher yield and greater purity than arc-discharge method	High synthesis temperature Contaminated with impurities
Carbon dots	Optical	Simple experimental setup Can tune the dimension of CDs by adjusting experiment parameters	Low yield
	Chemical	High yield Large scale production Inexpensive apparatus	Requires hash chemicals Environmentally unfriendly
	Thermal	Rapid process Can be prepared using simple apparatus such as domestic microwave oven Can be carried out without hash chemical	Uneven heating Large size distribution
Graphene	Mechanical exfoliation	Simple procedure High quality graphene	Low yield
	Liquid exfoliation	Large scale production	Difficult to control the number of layers and defects Difficult to remove residual solvents
	Chemical treatment	Large scale production	Requires harsh chemical Long duration
Nanodiamonds	Detonation	Can be prepared from precursors such as old munitions	Requires purification High synthesis temperature
	Pulsed laser	Can be carried out in water Environmentally friendly procedure	Resultant product contains impurities

**Table 3:** Summary of carbon allotrope-based OFS for RH sensing.

Sensing Material	Optical Fiber	Linear Detection Range (%)	Sensitivity (% RH^−1^)	Ref.
CNT	Etched FBG	20–90	31 pm	[147]
CNTs doped PMMA	Optical microfiber	45–80	0.3341 dBm	[149]
MWCNTs slurry	Optical microfiber	45–80	5.17 μW	[148]
CNT/PVA	Thin core fiber	70–86	0.4573 dB	[150]
rGO	Hollow core fiber	60–90	0.229 dB	[151]
rGO	Side polished fiber	70–95	0.31 dB	[134]
rGO	Microfiber resonator	30–50	0.0537 nm	[153]
rGO/PS	Optical microfiber	50.5–70.6 79.5–85.0	0.224 dB 4.118 dB	[152]
GO	Side polished fiber	32–85 85–97.6	0.145 nm 0.915 nm	[154]
GO	Side polished twin core fiber	40–75	2.720 nm	[155]
GO	Polarization maintaining fiber	60–77	0.349 dB	[156]
GO/PEI	Multimode fiber	20–70 70–90	0.317 nm 0.311 nm	[157]
GO	Tilted FBG	10–80	0.129 dB	[158]
GO	Tilted FBG	30–80	0.027 dB 18.5 pm	[159]
GO	Single mode fiber	30–60	0.104 dB 0.0272 nm	[160]
GO/PVA	Optical microfiber	40–60	0.0606 dBm	[161]
GO/PVA	Waist enlarged taper SMF	25–80	0.193 dB	[162]

**Table 4:** Summary of carbon allotrope-based OFS for temperature sensing.

Sensing Material	Optical Fiber	Linear Detection Range (° C)	Sensitivity (° C^−1^)	Ref.
rGO	Side polished fiber	−7.8–77	0.134 dB	[1]
rGO	Etched FBG	−100–300	33 pm	[164]
rGO	Suspended core hollow fiber	30–80	179.4 pm	[165]
Graphene	Optical microfiber	30–80	0.1018 dB	[166]
Graphene/PDMS	Microfiber ring resonator	30–60	0.544 dB	[167]
Graphene/Ag	Hollow core fiber	22–47	9.44 nm	[170]
PU/Graphene	FBG	25–60	6 pm	[171]
Graphene diaphragm	Fabry-Perot interferometer	20–60	352 nm	[172]
Graphene diaphragm	Fabry-Perot interferometer	500–510 1000–1008	1.56 nm 1.87 nm	[173]
Graphene diaphragm	FBG	27–77	13.31 pm	[168]
Graphene diaphragm	FBG	20–100	306.2 nm	[7]

**Table 5:** Summary of carbon allotrope-based OFS for pressure sensing.

Sensing Material	Optical Fiber	Linear Detection Range (kPa)	Sensitivity (nm kPa^−1^)	Ref.
Graphene diaphragm	Fabry-Perot interferometer	0–3.5	1096	[174]
Graphene diaphragm	Fabry-Perot interferometer	0–2.5	80	[175]
Graphene diaphragm	Fabry-Perot interferometer	0–13	65.71	[169]
Graphene diaphragm	Fabry-Perot interferometer	0–5	39.4	[176]
Graphene diaphragm	FBG	0–2	501.4	[7]
Graphene diaphragm	FBG	0–9.81	0.25	[168]

**Table 6:** Summary of carbon allotrope-based OFS for ethanol sensing.

Sensing Material	Optical Fiber	Detection Range (%)	LOD* (%)	Sensitivity	Ref.
Graphene	Tapered MMF	0–40	-	0.829 a.u. / %	[194]
GO	Plastic cladding silica fiber	10–80	5	-	[198]
GO	Tapered MMF	5–40	5	-	[199]
GO	FBG	0–80	10	-	[200]
GO	Tapered MMF	0–40	-	1.330 a.u. / %	[194]
SnO_2_/GO/PEI	MMF	0–100	20	-	[119]
Ag/rGO	MMF	0–100	1	-	[195]
CNTs	Tapered tip fiber	5–80	-	0.144 a.u. / %	[196]
CNTs	POF	20–100	0.2	-	[201]
CNTs	MMF	5–80	0.02	-	[133]

* Limit of Detection (LOD) in percentage concentration.

**Table 7:** Summary of carbon allotrope-based OFS for VOCs sensing.

Target Molecule	Sensing Material	Optical Fiber	Detection Range	LOD	Sensitivity*	Ref.
NH_3_	GO-MWCNTs	MMF	0–500 ppm	–	0.41 cts ppm^−1^	[202]
	GO	MMF	0–500 ppm	–	−0.32 cts ppm^−1^	[206]
	rGO	MMF	0–500 ppm	–	0.08 cts ppm^−1^	[202]
	Ag/GO	MMF	0–500 ppm	–	0.17 cts ppm^−1^	[202]
	MWCNTs	MMF	0–500 ppm	–	0.31 cts ppm^−1^	[207]
	SWCNTs	MMF	50–500 ppm	–	0.22 cts ppm^−1^	[208]
	Graphene	Optical microfiber	0–300 ppm	–	0.015 nm/ppm	[209]
	Graphene/PANI	Side polished fiber	0–1%	–	132.8 a.u./%	[210]
	Graphene	D-shaped fiber	0–1000 ppm	0.04 ppm	–	[211]
	Graphene	Microfiber Bragg grating	0–100 ppm	0.2 ppm	–	[212]
	Fe_3_O_4_ - graphene	SMF	1.5–150 ppm	7 ppb	–	[213]
	Graphene	Microfiber hybrid waveguide	0–360 ppm	0.3 ppm	–	[214]
CH_4_	Graphene/CNTs	MMF	10–100 ppm	–	0.3 nm/ppm	[215]
	PAA-NTs/PAH	PCF-LPG	0–3.5%	0.18%	1.078 nm/%	[216]
	SnO_2_/Graphene	Side polished fiber	0–55%	–	200 a.u./%	[205]
Ethanol	GO-MWCNTs	MMF	0–500 ppm	–	0.36 cts ppm^−1^	[203]
	GO	MMF	0–500 ppm	–	-0.26 cts ppm^−1^	[206]
	rGO	MMF	0–500 ppm	–	0.065 cts ppm^−1^	[217]
	MWCNTs	MMF	0–500 ppm	–	0.52 cts ppm^−1^	[218]
	SWCNTs	MMF	50–500 ppm	–	0.20 cts ppm^−1^	[208]
	CdA/SWCNTs	Fabry-Perot interferometer	–	–	0.5 10^−3^ / ppm	[204]
Methanol	GO-MWCNTs	MMF	0–500 ppm	–	0.23 cts ppm^−1^	[203]
	GO	MMF	0–500 ppm	–	−0.20 cts ppm^−1^	[206]
	rGO	MMF	0–500 ppm	–	0.038 cts ppm^−1^	[217]
	MWCNTs	MMF	0 – 500 ppm	–	0.14 cts ppm^−1^	[218]
	SWCNTs	MMF	50–500 ppm	–	0.01 cts ppm^−1^	[208]
Toluene	rGO	Side polished fiber	50–200 ppm	79 ppm	–	[219]
	CdA/SWCNTs	Fabry-Perot interferometer	–	–	1.3 10^−3^ / ppm	[204]
Xylene	Graphene	Microfiber Bragg grating	0–100 ppm	0.5 ppm	–	[212]
	CdA/SWCNTs	Fabry-Perot interferometer	–	–	3.2 10^−3^ / ppm	[204]

* cts ppm^−1^ = counts ppm^−1^, Limit of Detection (LOD).

**Table 8:** Advantages and limitations of different sensing methods for environmental and biological sensing applications.

Sensing Methods	Advantages	Limitations
Optical fiber [5]	Small size Long-term cost savings Multiplexing capabilities Distributed sensing capabilities Multifunctional sensing capabilities Large dynamic operating range Self-referencing Fast response time Resistant to electromagnetic interference Corrosion resistant Can be used in harsh environments Versatile sensing platform Miniaturized and portable sensor Feasible on-site or in-situ monitoring	Requires custom design of the interrogation system Cross sensitivities to physical parameters
Electrochemical (e.g., potential, current, conductivity, resistance [230])	Chemical signal converts to electrical signal that can be used immediately for electrical hardware Fast response time Miniaturized and portable sensor Potential for mass production Feasible on-site or in-situ monitoring	Complicated sensor configuration Cross sensitivities to physical parameters
Acoustic (e.g., surface acoustic wave [231,232])	Low power consumption Small size Able to work without batteries	Cross sensitivities to physical parameters Requires costly electronic detection system
Microwave (e.g., microwave resonator [233,234])	Capable of non-contact sensing Miniaturization is easy due to high operating frequencies Easily linked with commercial mobile communication system / wireless sensing network Inherently stable because the resonant frequency is related to the physical dimension	Cross sensitivities to physical parameters Susceptible to electromagnetic interference Requires high degree of specialization Cost of sensor configuration increase with frequency Long wavelengths cause the achievable resolution is limited
Mass spectroscopy [235]	Ultra-high sensitivity and selectivity High precision and accuracy	Expensive setup Bulky Requires high degree of specialization Tedious sample preparation

## References

[1] Zhang J., Liao G., Jin S., Cao D., Wei Q., Lu H., Yu J., Cai X., Tan S., Xiao Y. (2014). All-fiber-optic temperature sensor based on reduced graphene oxide. Laser Phys. Lett..

[2] Wang Q., Wang B.-T. (2018). Surface plasmon resonance biosensor based on graphene oxide/silver coated polymer cladding silica fiber. Sens. Actuators B Chem..

[3] Chan K.K., Yap S.H.K., Yong K.-T. (2018). Biogreen Synthesis of Carbon Dots for Biotechnology and Nanomedicine Applications. Nano Micro Lett..

[4] Chien Y.-H., Chan K.K., Yap S.H.K., Yong K.-T. (2018). NIR-responsive nanomaterials and their applications; upconversion nanoparticles and carbon dots: A perspective. J. Chem. Technol. Biotechnol..

[5] Grattan K.T.V. (1999). Optical Fiber Sensor Technology Chemical and Environmental Sensing.

[6] Wu Y., Yao B., Yu C., Rao Y. (2018). Optical Graphene Gas Sensors Based on Microfibers: A Review. Sensors.

[7] Dong N., Wang S., Jiang L., Jiang Y., Wang P., Zhang L. (2018). Pressure and Temperature Sensor Based on Graphene Diaphragm and Fiber Bragg Gratings. IEEE Photonics Technol. Lett..

[8] Zhao Y., Li X.-g., Zhou X., Zhang Y.-n. (2016). Review on the graphene based optical fiber chemical and biological sensors. Sens. Actuators B Chem..

[9] Consales M., Cutolo A., Penza M., Aversa P., Giordano M., Cusano A. (2008). Fiber optic chemical nanosensors based on engineered single-walled carbon nanotubes. J. Sens..

[10] Iijima S. (1991). Helical microtubules of graphitic carbon. Nature.

[11] Iijima S., Ichihashi T. (1993). Single-shell carbon nanotubes of 1-nm diameter. Nature.

[12] Niyogi S., Hamon M.A., Hu H., Zhao B., Bhowmik P., Sen R., Itkis M.E., Haddon R.C. (2002). Chemistry of Single-Walled Carbon Nanotubes. Acc. Chem. Res..

[13] Bahr J.Y., Yang J., Kosynkin D.V., Bronikowski M.J., Smalley R.E., Tour J.M. (2001). Functionalization of carbon nanotubes by electrochemical reduction of aryl diazonium salts: A bucky paper electrode. J. Am. Chem. Soc.

[14] Agnihotri S., Mota J.P., Rostam-Abadi M., Rood M.J. (2005). Structural characterization of single-walled carbon nanotube bundles by experiment and molecular simulation. Langmuir.

[15] Gao S., Zhuang R.C., Zhang J., Liu J.W., Mäder E. (2010). Glass fibers with carbon nanotube networks as multifunctional sensors. Adv. Funct. Mater..

[16] Lee R.S., Kim H.J., Fischer J., Thess A., Smalley R.E. (1997). Conductivity enhancement in single-walled carbon nanotube bundles doped with K and Br. Nature.

[17] Claye A.S., Fischer J.E., Huffman C.B., Rinzler A.G., Smalley R.E. (2000). Solid-state electrochemistry of the Li single wall carbon nanotube system. J. Electrochem. Soc..

[18] Hirsch A. (2002). Functionalization of Single-Walled Carbon Nanotubes. Angew. Chem. Int. Ed..

[19] Kamaras K., Itkis M.E., Hu H., Zhao B., Haddon R.C. (2003). Covalent Bond Formation to a Carbon Nanotube Metal. Science.

[20] Xu X., Ray R., Gu Y., Ploehn H.J., Gearheart L., Raker K., Scrivens W.A. (2004). Electrophoretic analysis and purification of fluorescent single-walled carbon nanotube fragments. J. Am. Chem. Soc..

[21] Ponomarenko L.A., Schedin F., Katsnelson M.I., Yang R., Hill E.W., Novoselov K.S., Geim A.K. (2008). Chaotic Dirac billiard in graphene quantum dots. Science.

[22] Jiang J., He Y., Li S., Cui H. (2012). Amino acids as the source for producing carbon nanodots: Microwave assisted one-step synthesis, intrinsic photoluminescence property and intense chemiluminescence enhancement. Chem. Commun..

[23] Hsu P.-C., Chang H.-T. (2012). Synthesis of high-quality carbon nanodots from hydrophilic compounds: Role of functional groups. Chem. Commun..

[24] Salinas-Castillo A., Ariza-Avidad M., Pritz C., Camprubí-Robles M., Fernández B., Ruedas-Rama M.J., Megia-Fernández A., Lapresta-Fernández A., Santoyo-Gonzalez F., Schrott-Fischer A. (2013). Carbon dots for copper detection with down and upconversion fluorescent properties as excitation sources. Chem. Commun..

[25] Hu S.-L., Niu K.-Y., Sun J., Yang J., Zhao N.-Q., Du X.-W. (2009). One-step synthesis of fluorescent carbon nanoparticles by laser irradiation. J. Mater. Chem..

[26] Dong Y., Wang R., Li H., Shao J., Chi Y., Lin X., Chen G. (2012). Polyamine-functionalized carbon quantum dots for chemical sensing. Carbon.

[27] Sun D., Ban R., Zhang P.-H., Wu G.-H., Zhang J.-R., Zhu J.-J. (2013). Hair fiber as a precursor for synthesizing of sulfur-and nitrogen-co-doped carbon dots with tunable luminescence properties. Carbon.

[28] Xu Q., Pu P., Zhao J., Dong C., Gao C., Chen Y., Chen J., Liu Y., Zhou H. (2015). Preparation of highly photoluminescent sulfur-doped carbon dots for Fe (III) detection. J. Mater. Chem. A.

[29] Shan X., Chai L., Ma J., Qian Z., Chen J., Feng H. (2014). B-doped carbon quantum dots as a sensitive fluorescence probe for hydrogen peroxide and glucose detection. Analyst.

[30] Jiang G., Jiang T., Li X., Wei Z., Du X., Wang X. (2014). Boronic acid functionalized N-doped carbon quantum dots as fluorescent probe for selective and sensitive glucose determination. Mater. Res. Express.

[31] Pan L., Sun S., Zhang A., Jiang K., Zhang L., Dong C., Huang Q., Wu A., Lin H. (2015). Truly fluorescent excitation-dependent carbon dots and their applications in multicolor cellular imaging and multidimensional sensing. Adv. Mater..

[32] Li X., Zhang S., Kulinich S.A., Liu Y., Zeng H. (2014). Engineering surface states of carbon dots to achieve controllable luminescence for solid-luminescent composites and sensitive Be 2+ detection. Sci. Rep..

[33] Chien Y.H., Chan K.K., Anderson T., Kong K.V., Ng B.K., Yong K.T. (2019). Advanced Near-Infrared Light-Responsive Nanomaterials as Therapeutic Platforms for Cancer Therapy. Adv. Ther..

[34] Sun Y.-P., Zhou B., Lin Y., Wang W., Fernando K.S., Pathak P., Meziani M.J., Harruff B.A., Wang X., Wang H. (2006). Quantum-sized carbon dots for bright and colorful photoluminescence. J. Am. Chem. Soc..

[35] Wang H., Sun C., Chen X., Zhang Y., Colvin V.L., Rice Q., Seo J., Feng S., Wang S., William W.Y. (2017). Excitation wavelength independent visible color emission of carbon dots. Nanoscale.

[36] Wang C., Xu Z., Cheng H., Lin H., Humphrey M.G., Zhang C. (2015). A hydrothermal route to water-stable luminescent carbon dots as nanosensors for pH and temperature. Carbon.

[37] Yu P., Wen X., Toh Y.-R., Tang J. (2012). Temperature-dependent fluorescence in carbon dots. J. Phys. Chem. C.

[38] Meng X., Chang Q., Xue C., Yang J., Hu S. (2017). Full-colour carbon dots: From energy-efficient synthesis to concentration-dependent photoluminescence properties. Chem. Commun..

[39] Jia X., Li J., Wang E. (2012). One-pot green synthesis of optically pH-sensitive carbon dots with upconversion luminescence. Nanoscale.

[40] Panwar N., Soehartono A.M., Chan K.K., Zeng S., Xu G., Qu J., Coquet P., Yong K.-T., Chen X. (2019). Nanocarbons for biology and medicine: Sensing, imaging, and drug delivery. Chem. Rev..

[41] Novoselov K.S., Geim A.K., Morozov S.V., Jiang D., Zhang Y., Dubonos S.V., Grigorieva I.V., Firsov A.A. (2004). Electric field effect in atomically thin carbon films. Science.

[42] Han T.-H., Lee Y., Choi M.-R., Woo S.-H., Bae S.-H., Hong B.H., Ahn J.-H., Lee T.-W. (2012). Extremely efficient flexible organic light-emitting diodes with modified graphene anode. Nat. Photonics.

[43] Pumera M. (2011). Graphene-based nanomaterials for energy storage. Energy Environ. Sci..

[44] Bonaccorso F., Colombo L., Yu G., Stoller M., Tozzini V., Ferrari A.C., Ruoff R.S., Pellegrini V. (2015). Graphene, related two-dimensional crystals, and hybrid systems for energy conversion and storage. Science.

[45] Shen H., Zhang L., Liu M., Zhang Z. (2012). Biomedical applications of graphene. Theranostics.

[46] Yang C., Chan K.K., Xu G., Yin M., Lin G., Wang X., Lin W.-J., Birowosuto M.D., Zeng S., Ogi T. (2018). Biodegradable Polymer-Coated Multifunctional Graphene Quantum Dots for Light-Triggered Synergetic Therapy of Pancreatic Cancer. ACS Appl. Mater. Interfaces.

[47] Hong G., Diao S., Antaris A.L., Dai H. (2015). Carbon Nanomaterials for Biological Imaging and Nanomedicinal Therapy. Chem. Rev..

[48] Liu J., Liu Z., Barrow C.J., Yang W. (2015). Molecularly engineered graphene surfaces for sensing applications: A review. Anal. Chim. Acta.

[49] Huang Y., Dong X., Shi Y., Li C.M., Li L.-J., Chen P. (2010). Nanoelectronic biosensors based on CVD grown graphene. Nanoscale.

[50] Kwak Y.H., Choi D.S., Kim Y.N., Kim H., Yoon D.H., Ahn S.-S., Yang J.-W., Yang W.S., Seo S. (2012). Flexible glucose sensor using CVD-grown graphene-based field effect transistor. Biosens. Bioelectron..

[51] Ling X., Xie L., Fang Y., Xu H., Zhang H., Kong J., Dresselhaus M.S., Zhang J., Liu Z. (2009). Can graphene be used as a substrate for Raman enhancement?. Nano Lett..

[52] Dresselhaus M.S., Jorio A., Hofmann M., Dresselhaus G., Saito R. (2010). Perspectives on carbon nanotubes and graphene Raman spectroscopy. Nano Lett..

[53] Qiu C., Zhou H., Yang H., Chen M., Guo Y., Sun L. (2011). Investigation of n-layer graphenes as substrates for Raman enhancement of crystal violet. J. Phys. Chem. C.

[54] Ju L., Geng B., Horng J., Girit C., Martin M., Hao Z., Bechtel H.A., Liang X., Zettl A., Shen Y.R. (2011). Graphene plasmonics for tunable terahertz metamaterials. Nat. Nanotechnol..

[55] Fei Z., Andreev G.O., Bao W., Zhang L.M., McLeod A.S., Wang C., Stewart M.K., Zhao Z., Dominguez G., Thiemens M. (2011). Infrared nanoscopy of Dirac plasmons at the graphene–SiO2 interface. Nano Lett..

[56] Wallace P.R. (1947). The band theory of graphite. Phys. Rev..

[57] Neto A.C., Guinea F., Peres N.M., Novoselov K.S., Geim A.K. (2009). The electronic properties of graphene. Rev. Mod. Phys..

[58] Marini A., Silveiro I.n., García de Abajo F.J. (2015). Molecular sensing with tunable graphene plasmons. ACS Photonics.

[59] Shang J., Ma L., Li J., Ai W., Yu T., Gurzadyan G.G. (2012). The origin of fluorescence from graphene oxide. Sci. Rep..

[60] Hernaez M., Zamarreño C., Melendi-Espina S., Bird L., Mayes A., Arregui F. (2017). Optical fibre sensors using graphene-based materials: A review. Sensors.

[61] Ruan Y., Ding L., Duan J., Ebendorff-Heidepriem H., Monro T.M. (2016). Integration of conductive reduced graphene oxide into microstructured optical fibres for optoelectronics applications. Sci. Rep..

[62] Zhang Y., Rhee K.Y., Hui D., Park S.-J. (2018). A critical review of nanodiamond based nanocomposites: Synthesis, properties and applications. Compos. Part B: Eng..

[63] Shenderova O., Zhirnov V., Brenner D. (2002). Carbon nanostructures. Crit. Rev. Solid State Mater. Sci..

[64] Greiner N.R., Phillips D., Johnson J., Volk F. (1988). Diamonds in detonation soot. Nature.

[65] Mochalin V.N., Neitzel I., Etzold B.J., Peterson A., Palmese G., Gogotsi Y. (2011). Covalent incorporation of aminated nanodiamond into an epoxy polymer network. ACS Nano.

[66] Liang Y., Ozawa M., Krueger A. (2009). A general procedure to functionalize agglomerating nanoparticles demonstrated on nanodiamond. ACS Nano.

[67] Pentecost A., Gour S., Mochalin V., Knoke I., Gogotsi Y. (2010). Deaggregation of nanodiamond powders using salt-and sugar-assisted milling. ACS Appl. Mater. Interfaces.

[68] Morita Y., Takimoto T., Yamanaka H., Kumekawa K., Morino S., Aonuma S., Kimura T., Komatsu N. (2008). A Facile and Scalable Process for Size-Controllable Separation of Nanodiamond Particles as Small as 4 nm. Small.

[69] Plakhotnik T. (2017). Diamonds for quantum nano sensing. Curr. Opin. Solid State Mater. Sci..

[70] Plakhotnik T., Aman H., Chang H.-C. (2015). All-optical single-nanoparticle ratiometric thermometry with a noise floor of 0.3 K Hz− 1/2. Nanotechnology.

[71] Plakhotnik T., Doherty M.W., Cole J.H., Chapman R., Manson N.B. (2014). All-optical thermometry and thermal properties of the optically detected spin resonances of the NV–center in nanodiamond. Nano Lett..

[72] Aman H., Plakhotnik T. (2016). Accuracy in the measurement of magnetic fields using nitrogen-vacancy centers in nanodiamonds. Josa B.

[73] Ando Y., Iijima S. (1993). Preparation of carbon nanotubes by arc-discharge evaporation. Jpn. J. Appl. Phys. Part 2 Lett..

[74] Wang Y., Yeow J.T. (2009). A review of carbon nanotubes-based gas sensors. J. Sens..

[75] Mubarak N., Abdullah E., Jayakumar N., Sahu J. (2014). An overview on methods for the production of carbon nanotubes. J. Ind. Eng. Chem..

[76] Ebbesen T.W. (1997). Production and purification of carbon nanotubes. ChemInform.

[77] Li J., Lu Y., Ye Q., Cinke M., Han J., Meyyappan M. (2003). Carbon nanotube sensors for gas and organic vapor detection. Nano Lett..

[78] Gonçalves H., Jorge P.A., Fernandes J., da Silva J.C.E. (2010). Hg (II) sensing based on functionalized carbon dots obtained by direct laser ablation. Sens. Actuators B Chem..

[79] Li X., Wang H., Shimizu Y., Pyatenko A., Kawaguchi K., Koshizaki N. (2010). Preparation of carbon quantum dots with tunable photoluminescence by rapid laser passivation in ordinary organic solvents. Chem. Commun..

[80] Chang M.M.F., Ginjom I.R., Ngu-Schwemlein M., Ng S.M. (2016). Synthesis of yellow fluorescent carbon dots and their application to the determination of chromium (III) with selectivity improved by pH tuning. Microchim. Acta.

[81] Hu Y., Yang J., Tian J., Jia L., Yu J.-S. (2014). Waste frying oil as a precursor for one-step synthesis of sulfur-doped carbon dots with pH-sensitive photoluminescence. Carbon.

[82] Dutta P., Ghosh T., Kumar H., Jain T., Singh Y. (2015). Hydrothermal and solvothermal synthesis of carbon dots from chitosan-ethanol system. Asian Chitin J.

[83] Li M., Yu C., Hu C., Yang W., Zhao C., Wang S., Zhang M., Zhao J., Wang X., Qiu J. (2017). Solvothermal conversion of coal into nitrogen-doped carbon dots with singlet oxygen generation and high quantum yield. Chem. Eng. J..

[84] Chan K.K., Yap S.H.K., Yong K. Solid State Carbon Dots-Based Sensor Using Optical Microfiber for Ferric Ion Detection. Proceedings of the 2019 IEEE International Conference on Sensors and Nanotechnology.

[85] Chan K.K., Yang C., Chien Y.-H., Panwar N., Yong K.-T. (2019). A Facile Synthesis of Label-Free Carbon Dots with Unique Selectivity-Tunable Characteristics for Ferric Ion Detection and Cellular Imaging Application. New J. Chem. Commun..

[86] Hernandez Y., Nicolosi V., Lotya M., Blighe F.M., Sun Z., De S., McGovern I., Holland B., Byrne M., Gun’Ko Y.K. (2008). High-yield production of graphene by liquid-phase exfoliation of graphite. Nat. Nanotechnol..

[87] Brodie B. (1855). Note sur un nouveau procédé pour la purification et la désagrégation du graphite. Ann. Chim. Phys..

[88] Staudenmaier L. (1898). Verfahren zur darstellung der graphitsäure. Ber. Der Dtsch. Chem. Ges..

[89] Hummers W.S., Offeman R.E. (1958). Preparation of graphitic oxide. J. Am. Chem. Soc..

[90] Jang J.W., Cho S., Moon G.h., Ihm K., Kim J.Y., Youn D.H., Lee S., Lee Y.H., Choi W., Lee K.H. (2012). Photocatalytic Synthesis of Pure and Water-Dispersible Graphene Monosheets. Chem. A Eur. J..

[91] Sutter P.W., Flege J.-I., Sutter E.A. (2008). Epitaxial graphene on ruthenium. Nat. Mater..

[92] Dedkov Y.S., Fonin M., Rüdiger U., Laubschat C. (2008). Rashba effect in the graphene/Ni (111) system. Phys. Rev. Lett..

[93] Li X., Wang X., Zhang L., Lee S., Dai H. (2008). Chemically derived, ultrasmooth graphene nanoribbon semiconductors. Science.

[94] Huang H., Chen W., Chen S., Wee A.T.S. (2008). Bottom-up growth of epitaxial graphene on 6H-SiC (0001). ACS Nano.

[95] Berger C., Song Z., Li X., Wu X., Brown N., Naud C., Mayou D., Li T., Hass J., Marchenkov A.N. (2006). Electronic confinement and coherence in patterned epitaxial graphene. Science.

[96] First P.N., de Heer W.A., Seyller T., Berger C., Stroscio J.A., Moon J.-S. (2010). Epitaxial graphenes on silicon carbide. Mrs Bull..

[97] Zangwill A., Vvedensky D.D. (2011). Novel growth mechanism of epitaxial graphene on metals. Nano Lett..

[98] Dolmatov V.Y. (2001). Detonation synthesis ultradispersed diamonds: Properties and applications. Russ. Chem. Rev..

[99] Shenderova O.A., Gruen D.M. (2012). Ultrananocrystalline Diamond: Synthesis, Properties and Applications.

[100] Danilenko V.V. (2004). On the history of the discovery of nanodiamond synthesis. Phys. Solid State.

[101] Mochalin V.N., Shenderova O., Ho D., Gogotsi Y. (2012). The properties and applications of nanodiamonds. Nat. Nanotechnol..

[102] Gruen D.M., Shenderova O.A., Vul A.Y. (2006). Synthesis, Properties and Applications of Ultrananocrystalline Diamond. Proceedings of the NATO ARW on Synthesis, Properties and Applications of Ultrananocrystalline Diamond.

[103] Krüger A., Kataoka F., Ozawa M.a.a., Fujino T., Suzuki Y., Aleksenskii A.E., Vul A.Y., Ōsawa E. (2005). Unusually tight aggregation in detonation nanodiamond: Identification and disintegration. Carbon.

[104] Ozawa M., Inaguma M., Takahashi M., Kataoka F., Krueger A., Ōsawa E. (2007). Preparation and behavior of brownish, clear nanodiamond colloids. Adv. Mater..

[105] Amans D., Chenus A.-C., Ledoux G., Dujardin C., Reynaud C., Sublemontier O., Masenelli-Varlot K., Guillois O. (2009). Nanodiamond synthesis by pulsed laser ablation in liquids. Diam. Relat. Mater..

[106] Bai P., Hu S., Zhang T., Sun J., Cao S. (2010). Effect of laser pulse parameters on the size and fluorescence of nanodiamonds formed upon pulsed-laser irradiation. Mater. Res. Bull..

[107] Kumar A., Lin P.A., Xue A., Hao B., Yap Y.K., Sankaran R.M. (2013). Formation of nanodiamonds at near-ambient conditions via microplasma dissociation of ethanol vapour. Nat. Commun..

[108] Petty M.C. (1996). Langmuir-Blodgett films an introduction.

[109] Rees N.D., James S.W., Tatam R.P., Ashwell G.J. (2002). Optical fiber long-period gratings with Langmuir–Blodgett thin-film overlays. Opt. Lett..

[110] Peterson I.R. (1990). Langmuir-Blodgett films. J. Phys. D Appl. Phys..

[111] Smietana M., Bock W.J., Szmidt J., Pickrell G.R. (2010). Nanocoating Enhanced Optical Fiber Sensors. Advances in Materials Science for Environmental and Nuclear Technology.

[112] Decher G., Hong J.D., Schmitt J. (1992). Buildup of ultrathin multilayer films by a self-assembly process: III. Consecutively alternating adsorption of anionic and cationic polyelectrolytes on charged surfaces. Thin Solid Films.

[113] Paul P.K., Hansda C., Hussain S.A. (2014). Layer-by-Layer Electrostatic Self-assembly Method: A Facile Approach of Preparing Nanoscale Molecular Thin Films. Invertis J. Sci. Technol..

[114] Gowri A., Sai V.V.R. (2016). Development of LSPR based U-bent plastic optical fiber sensors. Sens. Actuators B Chem..

[115] Lindgren E.B., Derbenev I.N., Khachatourian A., Chan H.-K., Stace A.J., Besley E. (2018). Electrostatic Self-Assembly: Understanding the Significance of the Solvent. J. Chem. Theory Comput..

[116] Verveniotis E., Kromka A., Ledinský M., Čermák J., Rezek B. (2011). Guided assembly of nanoparticles on electrostatically charged nanocrystalline diamond thin films. Nanoscale Res. Lett..

[117] Gonçalves H.M.R., Duarte A.J., Davis F., Higson S.P.J., Esteves da Silva J.C.G. (2012). Layer-by-layer immobilization of carbon dots fluorescent nanomaterials on single optical fiber. Anal. Chim. Acta.

[118] Alberto N., Vigário C., Duarte D., Almeida N.A.F., Gonçalves G., Pinto J.L., Marques P.A.A.P., Nogueira R., Neto V. (2015). Characterization of Graphene Oxide Coatings onto Optical Fibers for Sensing Applications. Mater. Today Proc..

[119] Hernaez M., Mayes A.G., Melendi-Espina S. (2018). Graphene Oxide in Lossy Mode Resonance-Based Optical Fiber Sensors for Ethanol Detection. Sensors.

[120] Martin P.M. (2010). Handbook of Deposition Technologies for Films and Coatings: Science, Applications and Technology.

[121] Yao B., Wu Y., Wang Z., Cheng Y., Rao Y., Gong Y., Chen Y., Li Y. (2013). Demonstration of complex refractive index of graphene waveguide by microfiber-based Mach–Zehnder interferometer. Opt. Express.

[122] Jeschkowski U., Niederwald H., Möhl W., Beier W., Disam J., Gohlke D., Lübbers K., Bach H., Krause D. (2003). Coating Technologies. Thin Films on Glass.

[123] Kashiwagi K., Yamashita S., Set S.Y. (2009). In-situ monitoring of optical deposition of carbon nanotubes onto fiber end. Opt. Express.

[124] Malagnino N., Pesce G., Sasso A., Arimondo E. (2002). Measurements of trapping efficiency and stiffness in optical tweezers. Opt. Commun..

[125] Jonáš A., Zemánek P. (2008). Light at work: The use of optical forces for particle manipulation, sorting, and analysis. Electrophoresis.

[126] Kashiwagi K., Yamashita S. (2009). Deposition of carbon nanotubes around microfiber via evanascent light. Opt. Express.

[127] Hermanson G.T., Hermanson G.T. (2013). Chapter 3 - The Reactions of Bioconjugation. Bioconjugate Techniques.

[128] Hartman F.C., Wold F. (1966). Bifunctional Reagents. Cross-Linking of Pancreatic Ribonuclease with a Diimido Ester1. J. Am. Chem. Soc..

[129] Avrameas S. (1969). Coupling of enzymes to proteins with glutaraldehyde: Use of the conjugates for the detection of antigens and antibodies. Immunochemistry.

[130] Hermanson G.T., Hermanson G.T. (2013). Chapter 4 - Zero-Length Crosslinkers. Bioconjugate Techniques.

[131] Yap S.H.K., Chan K.K., Chien Y., Yong K. Factors Influencing Metal Binding Efficiency at Solid/Liquid Interface: An Investigation for the Prediction of Heavy Metal Ion Sensing Performance. Proceedings of the 2019 IEEE International Conference on Sensors and Nanotechnology.

[132] Yap S.H.K., Chan K.K., Zhang G., Tjin S.C., Yong K.-T. (2019). Carbon Dot-functionalized Interferometric Optical Fiber Sensor for Detection of Ferric Ions in Biological Samples. ACS Appl. Mater. Interfaces.

[133] Shabaneh A.A., Girei S.H., Arasu P.T., Rashid S.A., Yunusa Z., Mahdi M.A., Paiman S., Ahmad M.Z., Yaacob M.H. (2014). Reflectance Response of Optical Fiber Coated With Carbon Nanotubes for Aqueous Ethanol Sensing. IEEE Photonics J..

[134] Xiao Y., Zhang J., Cai X., Tan S., Yu J., Lu H., Luo Y., Liao G., Li S., Tang J. (2014). Reduced graphene oxide for fiber-optic humidity sensing. Opt. Express.

[135] Azzuhri S.R.A., Amiri I.S., Zulkhairi A.S., Salim M.A.M., Razak M.Z.A., Khyasudeen M.F., Ahmad H., Zakaria R., Yupapin P. (2018). Application of graphene oxide based Microfiber-Knot resonator for relative humidity sensing. Results Phys..

[136] Kuo C.Y., Chan C.L., Gau C., Liu C., Shiau S.H., Ting J. (2007). Nano Temperature Sensor Using Selective Lateral Growth of Carbon Nanotube Between Electrodes. IEEE Trans. Nanotechnol..

[137] Choi S.H., Kim Y.L., Byun K.M. (2011). Graphene-on-silver substrates for sensitive surface plasmon resonance imaging biosensors. Opt. Express.

[138] Sharma A.K., Gupta B.D. (2005). On the sensitivity and signal to noise ratio of a step-index fiber optic surface plasmon resonance sensor with bimetallic layers. Opt. Commun..

[139] Zeng S., Hu S., Xia J., Anderson T., Dinh X.-Q., Meng X.-M., Coquet P., Yong K.-T. (2015). Graphene–MoS_2_ hybrid nanostructures enhanced surface plasmon resonance biosensors. Sens. Actuators B Chem..

[140] Prabowo B., Purwidyantri A., Liu K.-C. (2018). Surface plasmon resonance optical sensor: A review on light source technology. Biosensors.

[141] Ikeda S., Okamoto A. (2008). Hybridization-Sensitive On–Off DNA Probe: Application of the Exciton Coupling Effect to Effective Fluorescence Quenching. Chem. Asian J..

[142] Ji W.B., Tan Y.C., Lin B., Tjin S.C., Chow K.K. (2014). Nonadiabatically Tapered Microfiber Sensor With Ultrashort Waist. IEEE Photonics Technol. Lett..

[143] Ji W.B., Liu H.H., Tjin S.C., Chow K.K., Lim A. (2012). Ultrahigh Sensitivity Refractive Index Sensor Based on Optical Microfiber. IEEE Photonics Technol. Lett..

[144] An G., Li S., Cheng T., Yan X., Zhang X., Zhou X., Yuan Z. (2019). Ultra-stable D-shaped Optical Fiber Refractive Index Sensor with Graphene-Gold Deposited Platform. Plasmonics.

[145] Fu H., Zhang M., Ding J., Wu J., Zhu Y., Li H., Wang Q., Yang C. (2019). A high sensitivity D-type surface plasmon resonance optical fiber refractive index sensor with graphene coated silver nano-columns. Opt. Fiber Technol..

[146] Sridevi S., Vasu K.S., Jayaraman N., Asokan S., Sood A.K. (2014). Optical bio-sensing devices based on etched fiber Bragg gratings coated with carbon nanotubes and graphene oxide along with a specific dendrimer. Sens. Actuators B Chem..

[147] Shivananju B.N., Yamdagni S., Fazuldeen R., Kumar A.K.S., Nithin S.P., Varma M.M., Asokan S. (2014). Highly Sensitive Carbon Nanotubes Coated Etched Fiber Bragg Grating Sensor for Humidity Sensing. IEEE Sens. J..

[148] Mohamed H., Irawati N., Ahmad F., Ibrahim M.H., Ambran S., Rahman M.A.A., Harun S.W. (2017). Optical humidity sensor based on tapered fiber with multi-walled carbon nanotubes slurry. Indones. J. Electr. Eng. Comput. Sci..

[149] Isa N.M., Irawati N., Harun S.W., Ahmad F., Rahman H.A., Yusoff M.H.M. (2018). Multi-walled carbon nanotubes doped Poly(Methyl MethAcrylate) microfiber for relative humidity sensing. Sens. Actuators A Phys..

[150] Ma Q.F., Tou Z.Q., Ni K., Lim Y.Y., Lin Y.F., Wang Y.R., Zhou M.H., Shi F.F., Niu L., Dong X.Y. (2018). Carbon-nanotube/Polyvinyl alcohol coated thin core fiber sensor for humidity measurement. Sens. Actuators B Chem..

[151] Gao R., Lu D.-f., Cheng J., Jiang Y., Jiang L., Qi Z.-m. (2016). Humidity sensor based on power leakage at resonance wavelengths of a hollow core fiber coated with reduced graphene oxide. Sens. Actuators B Chem..

[152] Xing Z., Zheng Y., Yan Z., Feng Y., Xiao Y., Yu J., Guan H., Luo Y., Wang Z., Zhong Y. (2019). High-sensitivity humidity sensing of microfiber coated with three-dimensional graphene network. Sens. Actuators B Chem..

[153] Ahmad H., Rahman M.T., Sakeh S.N.A., Razak M.Z.A., Zulkifli M.Z. (2016). Humidity sensor based on microfiber resonator with reduced graphene oxide. Optik.

[154] Huang Y., Zhu W., Li Z., Chen G., Chen L., Zhou J., Lin H., Guan J., Fang W., Liu X. (2018). High-performance fibre-optic humidity sensor based on a side-polished fibre wavelength selectively coupled with graphene oxide film. Sens. Actuators B Chem..

[155] Chu R., Guan C., Bo Y., Shi J., Zhu Z., Li P., Yang J., Yuan L. (2019). All-optical graphene-oxide humidity sensor based on a side-polished symmetrical twin-core fiber Michelson interferometer. Sens. Actuators B Chem..

[156] Wang Y., Shen C., Lou W., Shentu F. (2016). Polarization-dependent humidity sensor based on an in-fiber Mach-Zehnder interferometer coated with graphene oxide. Sens. Actuators B Chem..

[157] Hernaez M., Acevedo B., Mayes A.G., Melendi-Espina S. (2019). High-performance optical fiber humidity sensor based on lossy mode resonance using a nanostructured polyethylenimine and graphene oxide coating. Sens. Actuators B Chem..

[158] Wang Y., Shen C., Lou W., Shentu F., Zhong C., Dong X., Tong L. (2016). Fiber optic relative humidity sensor based on the tilted fiber Bragg grating coated with graphene oxide. Appl. Phys. Lett..

[159] Jiang B., Bi Z., Hao Z., Yuan Q., Feng D., Zhou K., Zhang L., Gan X., Zhao J. (2019). Graphene oxide-deposited tilted fiber grating for ultrafast humidity sensing and human breath monitoring. Sens. Actuators B Chem..

[160] Liu S., Meng H., Deng S., Wei Z., Wang F., Tan C. (2018). Fiber Humidity Sensor Based on a Graphene-Coated Core-Offset Mach–Zehnder Interferometer. IEEE Sens. Lett..

[161] Mohamed H., Hussin N., Ahmad F., Ambran S., Harun S.W. Optical based relative humidity sensor using tapered optical fiber coated with graphene oxide. Proceedings of the AIP Conference.

[162] Weimin L., Youqing W., Changyu S., Fengying S. An optical fiber humidity sensor based on Mach-Zehnder interferometer coated with a composite film of graphene oxide and polyvinyl alcohol. Proceedings of the 15th International Conference on Optical Communications and Networks (ICOCN).

[163] Falkovsky L.A. Optical properties of graphene. Proceedings of the the International Conference on Theoretical Physics ‘DUBNA-NANO2008’.

[164] Vasu K.S., Asokan S., Sood A.K. (2016). Enhanced strain and temperature sensing by reduced graphene oxide coated etched fiber Bragg gratings. Opt. Lett..

[165] Chu R., Guan C., Bo Y., Shi J., Zhu Z., Li P., Yang J., Yuan L. (2019). Temperature Sensor in Suspended Core Hollow Fiber Covered With Reduced Graphene Oxide. IEEE Photonics Technol. Lett..

[166] Sun Q., Sun X., Jia W., Xu Z., Luo H., Liu D., Zhang L. (2016). Graphene-Assisted Microfiber for Optical-Power-Based Temperature Sensor. IEEE Photonics Technol. Lett..

[167] Wang M., Li D., Wang R., Zhu J., Ren Z. (2018). PDMS-assisted graphene microfiber ring resonator for temperature sensor. Opt. Quantum Electron..

[168] Ameen O.F., Younus M.H., Aziz M.S., Azmi A.I., Raja Ibrahim R.K., Ghoshal S.K. (2016). Graphene diaphragm integrated FBG sensors for simultaneous measurement of water level and temperature. Sens. Actuators A Phys..

[169] Cui Q., Thakur P., Rablau C., Avrutsky I., Cheng M.M. (2019). Miniature Optical Fiber Pressure Sensor With Exfoliated Graphene Diaphragm. IEEE Sens. J..

[170] Xu W., Yao J., Yang X., Shi J., Zhao J., Zhang C. (2016). Analysis of Hollow Fiber Temperature Sensor Filled with Graphene-Ag Composite Nanowire and Liquid. Sensors.

[171] Jasmi F., Azeman N.H., Bakar A.A.A., Zan M.S.D., Badri K.H., Su’ait M.S. (2018). Ionic Conductive Polyurethane-Graphene Nanocomposite for Performance Enhancement of Optical Fiber Bragg Grating Temperature Sensor. IEEE Access.

[172] Li C., Liu Q., Peng X., Fan S. (2015). Analyzing the temperature sensitivity of Fabry-Perot sensor using multilayer graphene diaphragm. Opt. Express.

[173] Li L., Feng Z., Qiao X., Yang H., Wang R., Su D., Wang Y., Bao W., Li J., Shao Z. (2015). Ultrahigh Sensitive Temperature Sensor Based on Fabry–Pérot Interference Assisted by a Graphene Diaphragm. IEEE Sens. J..

[174] Li C., Xiao J., Guo T., Fan S., Jin W. (2015). Interference characteristics in a Fabry–Perot cavity with graphene membrane for optical fiber pressure sensors. Microsyst. Technol..

[175] Li C., Xiao J., Guo T., Fan S., Jin W. (2015). Effects of graphene membrane parameters on diaphragm-type optical fibre pressure sensing characteristics. Mater. Res. Innov..

[176] Ma J., Jin W., Ho H.L., Dai J.Y. (2012). High-sensitivity fiber-tip pressure sensor with graphene diaphragm. Opt. Lett..

[177] Zheng B.-C., Yan S.-C., Chen J.-H., Cui G.-X., Xu F., Lu Y.-Q. (2015). Miniature optical fiber current sensor based on a graphene membrane. Laser Photonics Rev..

[178] Wang D., Fan S., Jin W. (2015). Graphene diaphragm analysis for pressure or acoustic sensor applications. Microsyst. Technol..

[179] Zhang Y., Wang F., Liu Z., Duan Z., Cui W., Han J., Gu Y., Wu Z., Jing Z., Sun C. (2017). Fiber-optic anemometer based on single-walled carbon nanotube coated tilted fiber Bragg grating. Opt. Express.

[180] Liu Y., Liang B., Zhang X., Hu N., Li K., Chiavaioli F., Gui X., Guo T. (2019). Plasmonic Fiber-Optic Photothermal Anemometers With Carbon Nanotube Coatings. J. Lightwave Technol..

[181] Liu Z., Wang F., Zhang Y., Jing Z., Peng W. (2019). Low-Power-Consumption Fiber-Optic Anemometer Based on Long-Period Grating With SWCNT Coating. IEEE Sens. J..

[182] Ruan Y., Simpson D.A., Jeske J., Ebendorff-Heidepriem H., Lau D.W.M., Ji H., Johnson B.C., Ohshima T., Afshar V.S., Hollenberg L. (2018). Magnetically sensitive nanodiamond-doped tellurite glass fibers. Sci. Rep..

[183] Wojciechowski A.M., Nakonieczna P., Mrózek M., Sycz K., Kruk A., Ficek M., Głowacki M., Bogdanowicz R., Gawlik W. (2019). Optical Magnetometry Based on Nanodiamonds with Nitrogen-Vacancy Color Centers. Materials.

[184] Rondin L., Tetienne J.P., Hingant T., Roch J.F., Maletinsky P., Jacques V. (2014). Magnetometry with nitrogen-vacancy defects in diamond. Rep. Prog. Phys..

[185] Ruan Y., Simpson D.A., Jeske J., Ebendorff-Heidepriem H., Lau D.W., Ji H., Johnson B.C., Ohshima T., Hollenberg L., Greentree A.D. (2016). Remote nanodiamond magnetometry. arXiv.

[186] Bai D., Capelli M., Huynh H., Ebendorff-Heidepriem H., Foster S., Greentree A.D., Gibson B.C. Hybrid Diamond-Glass Optical Fibres for Magnetic Sensing. Proceedings of the 26th International Conference on Optical Fiber Sensors.

[187] Yap S.H.K., Chien Y.-H., Tan R., bin Shaik Alauddin A.R., Ji W.B., Tjin S.C., Yong K.-T. (2018). An Advanced Hand-Held Microfiber-Based Sensor for Ultrasensitive Lead Ion Detection. ACS Sens..

[188] Wang C., Sun Y., Jin J., Xiong Z., Li D., Yao J., Liu Y. (2018). Highly selective, rapid-functioning and sensitive fluorescent test paper based on graphene quantum dots for on-line detection of metal ions. Anal. Methods.

[189] Ji W.B., Yap S.H.K., Panwar N., Zhang L.L., Lin B., Yong K.T., Tjin S.C., Ng W.J., Majid M.B.A. (2016). Detection of low-concentration heavy metal ions using optical microfiber sensor. Sens. Actuators B Chem..

[190] Abdulkhaleq Alwahib A., Fawzi Alhasan S., Yaacob M.H., Lim H.N., Adzir Mahdi M. (2020). Surface plasmon resonance sensor based on D-shaped optical fiber using fiberbench rotating wave plate for sensing pb ions. Optik.

[191] Yao B.C., Wu Y., Yu C.B., He J.R., Rao Y.J., Gong Y., Fu F., Chen Y.F., Li Y.R. (2016). Partially reduced graphene oxide based FRET on fiber-optic interferometer for biochemical detection. Sci. Rep..

[192] Lin Y., Dong X., Yang J., Maa H., Zu P., So P.L., Chan C.C. Detection of Ni^2+^ with optical fiber Mach-Zehnder interferometer coated with chitosan/MWCNT/PAA. Proceedings of the 16th International Conference on Optical Communications and Networks (ICOCN).

[193] Gonçalves H.M.R., Duarte A.J., Esteves da Silva J.C.G. (2010). Optical fiber sensor for Hg(II) based on carbon dots. Biosens. Bioelectron..

[194] Girei S.H., Shabaneh A.A., Ngee-Lim H., Hamidon M.N., Mahdi M.A., Yaacob M.H. (2015). Tapered optical fiber coated with graphene based nanomaterials for measurement of ethanol concentrations in water. Opt. Rev..

[195] Aziz A., Lim H.N., Girei S.H., Yaacob M.H., Mahdi M.A., Huang N.M., Pandikumar A. (2015). Silver/graphene nanocomposite-modified optical fiber sensor platform for ethanol detection in water medium. Sens. Actuators B Chem..

[196] Shabaneh A., Girei S., Arasu P., Mahdi M., Rashid S., Paiman S., Yaacob M. (2015). Dynamic Response of Tapered Optical Multimode Fiber Coated with Carbon Nanotubes for Ethanol Sensing Application. Sensors.

[197] Khalaf A.L., Shabaneh A.A.A., Yaacob M.H., Rashid S.A., Raja Othman R.N.I., Hussein M.Z. (2019). Chapter 10 - Carbon Nanotubes and Graphene Oxide Applications in Optochemical Sensors. Synthesis, Technology and Applications of Carbon Nanomaterials.

[198] Gao S.S., Qiu H.W., Zhang C., Jiang S.Z., Li Z., Liu X.Y., Yue W.W., Yang C., Huo Y.Y., Feng D.J. (2016). Absorbance response of a graphene oxide coated U-bent optical fiber sensor for aqueous ethanol detection. RSC Adv..

[199] Girei S.H., Shabaneh A.A., Lim H.M., Huang N.H., Mahdi M.A., Yaacob M.H. (2015). Absorbance response of graphene oxide coated on tapered multimode optical fiber towards liquid ethanol. J. Eur. Opt. Soc. Rapid Publ..

[200] Arasu P.T., Noor A.S., Shabaneh A.A., Yaacob M.H., Lim H.N., Mahdi M.A. (2016). Fiber Bragg grating assisted surface plasmon resonance sensor with graphene oxide sensing layer. Opt. Commun..

[201] Khalaf A.L., Arasu P.T., Lim H.N., Paiman S., Yusof N.A., Mahdi M.A., Yaacob M.H. (2017). Modified plastic optical fiber with CNT and graphene oxide nanostructured coatings for ethanol liquid sensing. Opt. Express.

[202] Kavinkumar T., Manivannan S. (2016). Uniform decoration of silver nanoparticle on exfoliated graphene oxide sheets and its ammonia gas detection. Ceram. Int..

[203] Kavinkumar T., Manivannan S. (2016). Synthesis, Characterization and Gas Sensing Properties of Graphene Oxide-Multiwalled Carbon Nanotube Composite. J. Mater. Sci. Technol..

[204] Consales M.C.A., Penza M., Aversa P., Veneri P.D., Giordano M., Cusano A. (2009). SWCNT nano-composite optical sensors for VOC and gas trace detection. Sens. Actuators B Chem..

[205] Zhang J.Y.D., Ding E.J., Xu S.C., Li Z.H., Wang X.X., Song F. (2017). Sensitization of an optical fiber methane sensor with graphene. Opt. Fiber Technol..

[206] Kavinkumar T., Sastikumar D., Manivannan S. (2015). Effect of functional groups on dielectric, optical gas sensing properties of graphene oxide and reduced graphene oxide at room temperature. RSC Adv..

[207] Manivannan S., Shobin L., Saranya A., Renganathan B., Sastikumar D., Park K.C. Carbon nanotubes coated fiber optic ammonia gas sensor. Proceedings of the Integrated Optics: Devices, Materials, and Technologies XV.

[208] Manivannan S., Saranya A.M., Renganathan B., Sastikumar D., Gobi G., Park K.C. (2012). Single-walled carbon nanotubes wrapped poly-methyl methacrylate fiber optic sensor for ammonia, ethanol and methanol vapors at room temperature. Sens. Actuators B Chem..

[209] Zhao Y., Zhang S.-Y., Wen G.-F., Han Z.-X. (2018). Graphene-based optical fiber ammonia gas sensor. Instrum. Sci. Technol..

[210] Khalaf A.L., Mohamad F.S., Rahman N.A., Lim H.N., Paiman S., Yusof N.A., Mahdi M.A., Yaacob M.H. (2017). Room temperature ammonia sensor using side-polished optical fiber coated with graphene/polyaniline nanocomposite. Opt. Mater. Express.

[211] Wu Y., Yao B.C., Zhang A.Q., Cao X.L., Wang Z.G., Rao Y.J., Gong Y., Zhang W., Chen Y.F., Chiang K.S. (2014). Graphene-based D-shaped fiber multicore mode interferometer for chemical gas sensing. Opt. Lett..

[212] Wu Y., Yao B., Zhang A., Rao Y., Wang Z., Cheng Y., Gong Y., Zhang W., Chen Y., Chiang K.S. (2014). Graphene-coated microfiber Bragg grating for high-sensitivity gas sensing. Opt. Lett..

[213] Pawar D., Rao B.V.B., Kale S.N. (2018). Fe_3_O_4_-decorated graphene assembled porous carbon nanocomposite for ammonia sensing: Study using an optical fiber Fabry–Perot interferometer. Analyst.

[214] Yao B.W.Y., Cheng Y., Zhang A., Gong Y., Rao Y.-J., Wang Z., Chen Y. (2014). All-optical Mach–Zehnder interferometric NH3 gas sensor based on graphene/microfiber hybrid waveguide. Sens. Actuators B Chem..

[215] Mishra S.K., Tripathi S.N., Choudhary V., Gupta B.D. (2015). Surface Plasmon Resonance-Based Fiber Optic Methane Gas Sensor Utilizing Graphene-Carbon Nanotubes-Poly(Methyl Methacrylate) Hybrid Nanocomposite. Plasmonics.

[216] Yang J., Che X., Shen R., Wang C., Li X., Chen W. (2017). High-sensitivity photonic crystal fiber long-period grating methane sensor with cryptophane-A-6Me absorbed on a PAA-CNTs/PAH nanofilm. Opt. Express.

[217] Kavinkumar T., Sastikumar D., Manivannan S. Reduced graphene oxide coated optical fiber for methanol and ethanol vapor detection at room temperature. .Proceedings of the SPIE/COS Photonics.

[218] Shobin L.R., Renganathan B., Sastikumar D., Park K.C., Manivannan S. (2014). Pure and Iso-Butyl Methyl Ketone Treated Multi-Walled Carbon Nanotubes for Ethanol and Methanol Vapor Sensing. IEEE Sens. J..

[219] Xiao Y., Yu J., Shun L., Tan S., Cai X., Luo Y., Zhang J., Dong H., Lu H., Guan H. (2016). Reduced graphene oxide for fiber-optic toluene gas sensing. Opt. Express.

[220] Qiu H.W.X., Xu S.C., Jiang S.Z., Li Z., Chen P.X., Gao S.S., Zhang C., Feng D.J. (2015). A novel graphene-based tapered optical fiber sensor for glucose detection. Appl. Surf. Sci..

[221] Jiang S., Li Z., Zhang C., Gao S., Li Z., Qiu H., Li C., Yang C., Liu M., Liu Y. (2017). A novel U-bent plastic optical fibre local surface plasmon resonance sensor based on a graphene and silver nanoparticle hybrid structure. J. Phys. D Appl. Phys..

[222] Sharma A.K., Gupta J. (2018). Graphene based chalcogenide fiber-optic evanescent wave sensor for detection of hemoglobin in human blood. Opt. Fiber Technol..

[223] Batumalay M. (2014). Tapered plastic optical fiber coated with single wall carbon nanotubes polyethylene oxide composite for measurement of uric acid concentration. Sens. Rev..

[224] Hu W.H.Y., Chen C., Liu Y., Guo T., Guan B.-O. (2018). Highly sensitive detection of dopamine using a graphene functionalized plasmonic fiber-optic sensor with aptamer conformational amplification. Sens. Actuators B Chem..

[225] Liu M.L., Chen B.B., Li C.M., Huang C.Z. (2019). Carbon dots: Synthesis, formation mechanism, fluorescence origin and sensing applications. Green Chem..

[226] Ham S.W., Hong H.P., Kim J.H., Min S.J., Min N.K. (2014). Effect of Oxygen Plasma Treatment on Carbon Nanotube-Based Sensors. J. Nanosci. Nanotechnol..

[227] Pandit S., Banerjee T., Srivastava I., Nie S., Pan D. (2019). Machine Learning-Assisted Array-Based Biomolecular Sensing Using Surface-Functionalized Carbon Dots. ACS Sens..

[228] Feder K.S., Westbrook P.S., Ging J., Reyes P.I., Carver G.E. (2003). In-fiber spectrometer using tilted fiber gratings. IEEE Photonics Technol. Lett..

[229] Wagener J.L., Strasser T.A., Pedrazzani J.R., DeMarco J., DiGiovanni D. Fiber grating optical spectrum analyzer tap. Proceedings of the 11th International Conference on Integrated Optics and Optical Fibre Communications, and 23rd European Conference on Optical Communications.

[230] Yogeswaran U., Chen S.-M. (2008). A Review on the Electrochemical Sensors and Biosensors Composed of Nanowires as Sensing Material. Sensors.

[231] Bo L., Xiao C., Hualin C., Mohammad M.A., Xiangguang T., Luqi T., Yi Y., Tianling R. (2016). Surface acoustic wave devices for sensor applications. J. Semicond..

[232] Go D.B., Atashbar M.Z., Ramshani Z., Chang H.-C. (2017). Surface acoustic wave devices for chemical sensing and microfluidics: A review and perspective. Anal Methods.

[233] Park J.-K., Kang T.-G., Kim B.-H., Lee H.-J., Choi H.H., Yook J.-G. (2018). Real-time Humidity Sensor Based on Microwave Resonator Coupled with PEDOT:PSS Conducting Polymer Film. Sci. Rep..

[234] Nyfors E. (2000). Industrial Microwave Sensors—A Review. Subsurf. Sens. Technol. Appl..

[235] Bruno T., Svoronos P. (2010). CRC Handbook of Basic Tables for Chemical Analysis.

